# Soluble immune checkpoints in lung cancer: linking prognostic signatures to immunotherapy response – a meta-analysis

**DOI:** 10.3389/fimmu.2026.1896427

**Published:** 2026-07-29

**Authors:** Larisa-Maria Rotariu, Teodora Alexa-Stratulat, Radu Tanasa, Mariana Pavel-Tanasa

**Affiliations:** 1 Grigore T. Popa University of Medicine and Pharmacy Iasi, Iasi, Romania; 2 Faculty of Physics, Alexandru Ioan Cuza University of Iasi, Iasi, Romania; 3 Laboratory of Immunology, St. Spiridon Emergency Clinical County Hospital, Iasi, Romania; 4 Advanced Research and Development Center for Experimental Medicine “Prof. Ostin C. Mungiu”—CEMEX, Grigore T. Popa University of Medicine and Pharmacy Iasi, Iasi, Romania

**Keywords:** immune checkpoint inhibitors, non-small cell lung cancer, overall survival, prognostic biomarker, progression-free survival, soluble PD-L1

## Abstract

**Introduction:**

Non-small cell lung cancer (NSCLC) remains one of the leading causes of cancer-related death, with most patients diagnosed at advanced, unresectable stages. Although immune-checkpoint inhibitors have transformed the therapeutic landscape, only a subset of patients achieves meaningful and sustained benefit. In this context, circulating soluble immune-checkpoints, particularly soluble PD-L1 (sPD-L1) have emerged as promising non-invasive biomarkers with potential prognostic and predictive value.

**Methods:**

To clarify their relevance, we conducted a systematic review and meta-analysis of studies published between 2015 and 2025, evaluating the association between serum levels of these biomarkers and clinical outcomes in NSCLC. A total of 20 studies including 1967 patients were analyzed, despite heterogeneity in study design and treatment regimens.

**Results:**

Lower baseline sPD-L1 levels consistently correlated with longer progression-free (PFS: random effect model with a pooled HR of 2.26 [95% CI: 1.77-2.87, p < 0.0001]) and overall survival (OS: random effect model with a pooled HR of 2.04 [95% CI 1.55-2.68, p < 0.0001]), whereas elevated concentrations were frequently observed in males, smokers, individuals with advanced disease, and those with liver metastases. Associations between soluble checkpoints and tumor histology, mutational status, or tissue PD-L1 expression varied widely across studies.

**Conclusions:**

Overall, our findings highlight baseline sPD-L1 with a median cut-off of 90 pg/mL as a promising prognostic biomarker for PFS in NSCLC, suggesting that dynamic monitoring combined with clinical and molecular parameters could enhance patient stratification. Prospective, standardized studies are needed to define optimal cut-offs and validate clinical utility across treatment settings.

## Graphical Abstract

**Figure:**
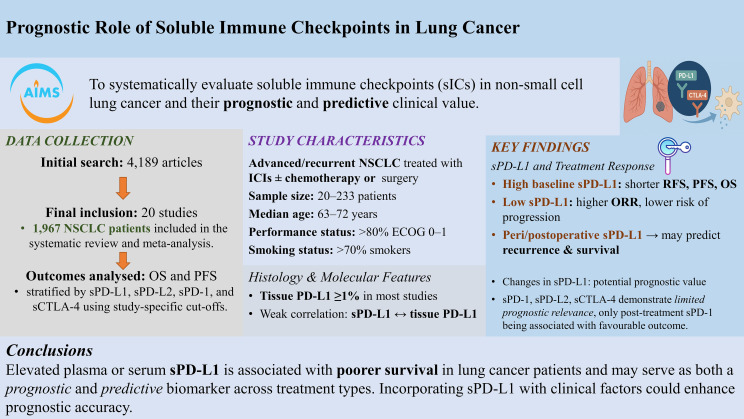
Overview of associations between baseline soluble PD-L1 (sPD-L1) levels and clinical outcomes in patients with non–small cell lung cancer (NSCLC). Patients with low baseline sPD-L1 levels demonstrated significantly longer progression-free survival (PFS) and overall survival (OS) compared with those with high sPD-L1 levels.

## 1 Introduction

Non-small cell lung cancer (NSCLC) remains one of the leading causes of cancer-related deaths, most cases being diagnosed in advanced inoperable stages. Therefore, the use of effective treatment regimens is warranted (1). Over the past few years, immune checkpoint inhibitors (ICIs) have proven to be potentially effective therapeutically, significantly transforming the field of oncology, especially in the management of lung cancer (2, 3). Response rates to immunotherapy in NSCLC are estimated at 20–30%, highlighting that a substantial proportion of patients derive limited clinical benefit. The biological mechanisms behind the divergent outcomes observed in morphologically similar lung tumors remain incompletely understood, with one potential factor being the scarcity of activated immune cells within the tumor microenvironment (TME) (4). While the clinical trial KEYNOTE-001 established a PD-L1 tissue expression cut-off of 50%, another study specifically enrolled patients with a tumor proportion score ≥50% who received either an anti-PD-1 antibody (Pembrolizumab) or chemotherapy. In this last cohort, the objective response rate was markedly higher in the pembrolizumab group [44.8%; 95% CI: 36.8–53.0] compared with the chemotherapy group [27.8%; 95% CI: 20.8–35.7] (5). Therefore, the identification of predictive and/or prognostic biomarkers for immunotherapy represents a valuable strategy in NSCLC treatment (6). Beyond their clinical utility, predictive biomarkers provide important insights into the mechanisms regulating anti-tumor immunity and immune evasion. A better understanding of the dynamic interactions between the immune system and the tumor microenvironment is essential for improving patient stratification and advancing precision immunotherapy in NSCLC.

PD-L1, also referred to as CD274 or B7-H1, is a transmembrane protein expressed on the surface of tumor cells and antigen-presenting immune cells. PD-1 is the receptor for PD-L1 and is expressed by T and B lymphocytes as well as myeloid cells. PD-L1 engages PD-1, and the resulting ligand–receptor interaction contributes to tumor immune escape by promoting the proliferation of cancer cells (7). The PD-1/PD-L1 immune checkpoint represents a central mechanism of peripheral immune regulation, controlling T-cell activation and effector function. Dysregulation of this pathway promotes immune tolerance within the tumor microenvironment and constitutes the biological rationale for immune checkpoint blockade. Tumor PD-L1 expression, widely accepted and recognized as standard biomarker, can predict the benefit from immunotherapy, being concurrently questionable and insufficient (8, 9). For instance, a PD-L1 negative patient may experience therapeutic benefit from treatment with ICIs and a high PD-L1 expression does not invariably correlate with a beneficial therapeutic outcome (10, 11). Furthermore, PD-L1 analysis is challenging due to intra-tumoral heterogeneity and the quality of the tissue material used, both of which are potential sources of error (9). Hence, there is a mandatory need for immunotherapy efficacy biomarkers that are accessible, easy to use and that can be monitored in clinical practice (6).

Soluble biomarkers can be easily assessed throughout the course of lung cancer (from diagnosis through disease progression), allowing for dynamic surveillance (12). Among these, soluble immune checkpoint molecules are circulating forms of immune checkpoint proteins that retain biological activity and may modulate anti-tumor immunity through interactions with ligands or receptors within the tumor microenvironment. Consequently, they may reflect systemic immune activation and the evolving immunological landscape during cancer progression and treatment. Consequently, they may provide complementary information regarding host immune status beyond conventional tissue-based biomarkers. Soluble immune checkpoint molecules have therefore attracted increasing interest as minimally invasive biomarkers with potential prognostic and predictive value across several malignancies, including NSCLC (13). One of the most extensively investigated molecules is soluble PD-L1 (sPD-L1), a circulating form of PD-L1 that can be quantified using non-invasive assays, such as ELISA, Luminex, or chemiluminescence. Importantly, sPD-L1 remains biologically active, exerting immunosuppressive effects by inhibiting T-cell activation and function (7, 14). Despite growing evidence supporting the clinical relevance of soluble immune checkpoints, their prognostic significance and association with therapeutic outcomes remain inconsistent across published studies. To address this gap, we performed a systematic review and meta-analysis to comprehensively evaluate the role of circulating soluble immune checkpoint molecules, with a particular focus on sPD-L1, as prognostic and predictive biomarkers in patients with NSCLC treated with immune checkpoint inhibitors. Furthermore, we sought to clarify their relationship with survival outcomes and clinicopathological characteristics, thereby contributing to a better understanding of the immunological determinants of response to cancer immunotherapy.

## 2 Materials and methods

### 2.1 Search strategy and inclusion criteria

This systematic review was performed following the Preferred Reporting Items for Systematic Reviews and Meta-Analyses (PRISMA) statement. On April 2025 we performed a systematic literature search of PubMed, Central Cochrane, EMBASE and Scopus by searching the terms: [“non-small cell lung cancer” OR “NSCLC”] AND [“soluble immune checkpoints” OR “soluble immune checkpoint molecules” OR “soluble PD-L1” OR “soluble PD-1” OR “soluble CTLA-4” OR “soluble PD-L2”] AND “survival” in the last 10 years (01.01.2015-30.04.2025). Studies were considered eligible if they reported individual patient data or Kaplan-Meier curves for overall survival (OS) and/or progression-free survival (PFS). The reference lists of original reports already published were also analyzed to identify other potential studies. The study’s eligibility criteria included: (i) studies including NSCLC patients; (ii) adult patients aged 18 years or more; (iii) primary endpoints: progression-free survival (PFS) and overall survival (OS); (iv) association of baseline soluble immune-checkpoint (sICP) values with PFS or OS; (v) cut-off values defined to differentiate between patients with low and high baseline levels of sICPs. Case reports or small case series (below 10 patients), editorials, reviews and articles not in English language were excluded from these analyses, and only retrospective or prospective cohort studies or randomized-control trials were kept.

### 2.2 Data Extraction and quality Assessment

Two investigators (LR and MPT) independently performed the title and abstract screening, full-text review, and data extraction. Any discrepancies were resolved through discussion and consensus. Data were extracted using a standardized form and included the following study characteristics: title, author, journal, year of publication, inclusion timeframe, region, study design, sample size, and treatment details. Treatment-related information included the type of immunotherapy, radiotherapy, chemotherapy, supportive therapy, or surgical intervention. Extracted data also covered primary and secondary outcomes, endpoints (progression-free survival (PFS) and overall survival (OS)), median follow-up, and cut-off values for sICPs. Patient-level information comprised gender, age, primary tumor histology, mutational gene status, smoking status, ECOG performance status, NSCLC stage, and resection status. Based on the cut-off values of sPD-L1, sPD-L2, sPD-1, and sCTLA-4 reported in each study included in this review, PFS and OS values were further extracted separately for the low and high sPD-L1 groups, as well as information on the methods used for determining soluble IPC levels and kit number (**Supplementary Tables 1**, **2**). In studies or cohorts comprising multiple types of neoplasms, the subset of patients with NSCLC and their corresponding data were manually extracted.

### 2.3 Statistical analysis

All statistical analyses were performed using SPSS v27, GraphPad Prism, and Python. Hazard ratios (HRs) for progression-free survival (PFS) and overall survival (OS) were extracted from the selected studies. In two studies, PFS was considered a surrogate marker for relapse-free survival (RFS). Pooled cut-off values are presented as median values with 95% confidence intervals (CIs), and survival outcomes (PFS and OS) were analyzed in patients stratified by low versus high sICP status.

Forest plots were generated to visualize the HRs for PFS and OS across the included studies. An HR greater than 1 indicated that patients with low baseline levels of sPD-L1 had poorer OS or PFS. The heterogeneity of fixed-effect and random-effects models was assessed using the I² statistic. Potential publication bias was visually evaluated using funnel plots for asymmetry and confirmed with Egger’s test. A threshold of p value below 0.05 was applied to determine statistical significance, and all reported findings met this criterion.

## 3 Results

### 3.1 Study selection

**Figure 1:**
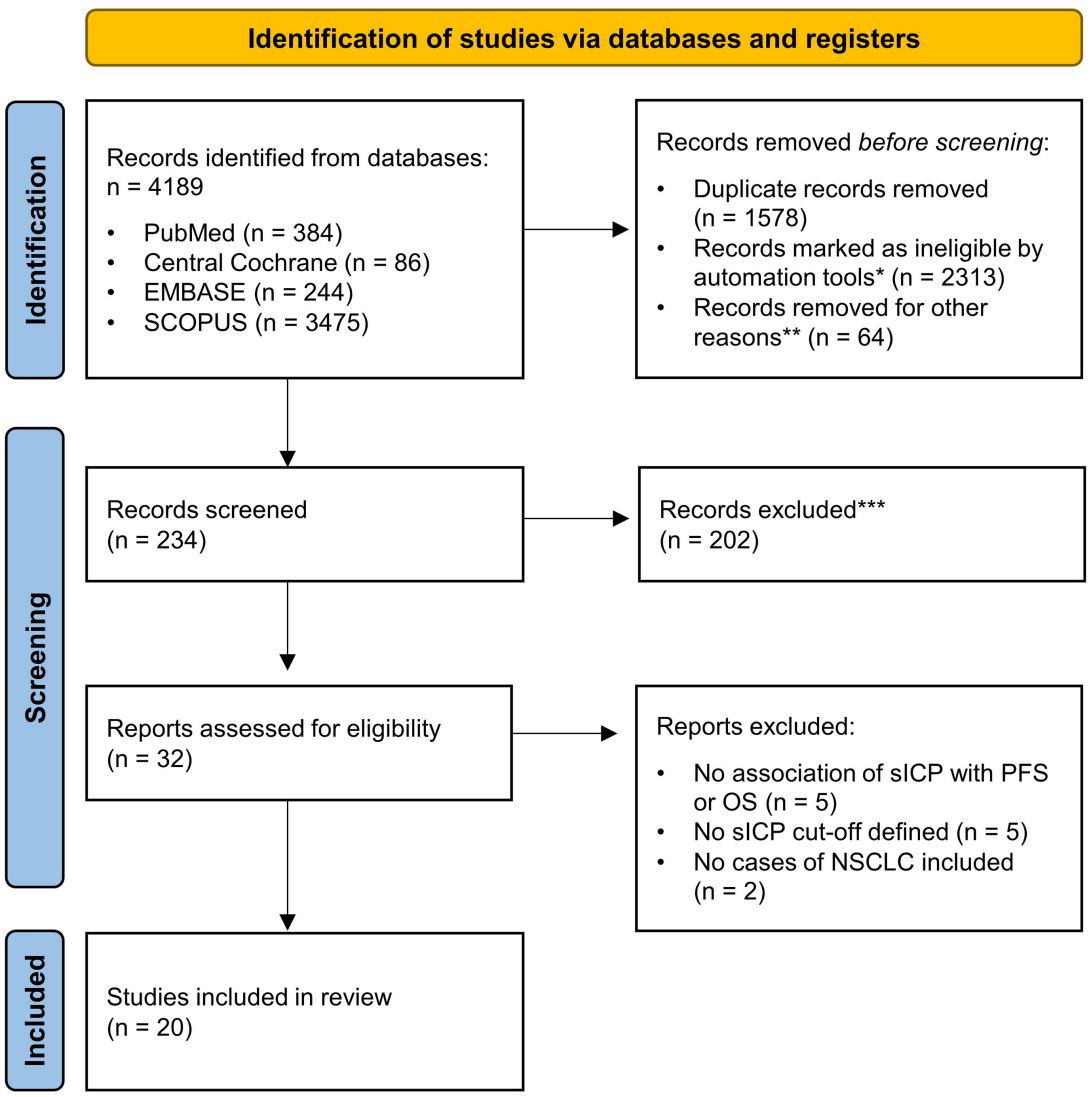
PRISMA diagram of the study selection process. *Records marked as ineligible by automation tools – records automatically classified as unlikely to meet the predefined eligibility criteria (including soluble immune checkpoint assessment, clinical outcomes, and study design requirements) before manual title and abstract screening. **Records removed for other reasons – records excluded during the initial database processing step based on predefined search restrictions and database filters (e.g., non-human studies, non-eligible publication types, and records outside the predefined date range). ***Records excluded after title and abstract screening - records were excluded because they lacked evidence of an association between soluble immune checkpoints and clinical outcomes (OS/PFS) based on title and abstract screening, or due to assessment of non-plasma/serum soluble immune checkpoint forms (e.g., exosome PD-L1), evaluation of soluble immune checkpoints in non-blood biological fluids, or unrelated biomarkers/targets.

The study selection process is summarized in **Figure 1**. In total, 4, 189 papers were identified through the literature search. After excluding duplicates, review articles, abstracts, animal studies, and book chapters, 234 articles remained for screening. Following title and abstract screening, 204 papers were excluded. During full-text screening, 12 articles were further excluded for the following reasons: no association of soluble immune-checkpoints with PFS or OS (n = 5), no sICP cut-off defined (n = 5), and no NSCLC cases included (n = 2). Finally, 20 studies involving NSCLC patients, comprising a total of 1, 967 individuals, met the inclusion criteria and were included in this systematic review and meta-analysis (**Figure 1**). Details of the included studies are presented in **Supplementary Table 2**. Overall survival (OS) and progression-free survival (PFS) were subcategorized according to sPD-L1, sPD-L2, sPD-1, and sCTLA-4 values, using the cut-off values reported in the respective studies.

### 3.2 Pre- and post-treatment levels of sPD-L1 and their association with different clinicopathological characteristics and clinical responses

#### 3.2.1 Study characteristics

Eleven original papers included in the review enrolled only NSCLC patients treated with immunotherapy monotherapy or combination therapy with chemotherapy. In addition, three other studies included NSCLC patients who received ICIs alongside individuals diagnosed with other types of cancer (15–17). One study included lung cancer patients receiving different treatment approaches, (chemotherapy, radiotherapy, targeted therapy, anti-PD-1 monoclonal antibody therapy, combination therapy, palliative care or follow-up) (6). Moreover, two articles enrolled NSCLC patients treated with chemotherapy only or in combination with radiotherapy (18, 19). Last, but not least, three original papers recruited patients who subsequently underwent surgery, with plasma sPD-L1 measured either perioperatively or solely postoperatively (9, 20, 21).

#### 3.2.2 Patients’ characteristics

The number of patients included in these studies varied, ranging from small sample sizes [e.g., n = 20 (22);] to larger cohorts [e.g., n = 189 (23); n = 233 (24)]. The median age of the included patients ranged between 68 and 72 years, with two cohorts reporting a median age of 63 and 65 years (2, 25). The majority of the research papers comprised patients with good performance status, with over 80% of individuals in two cohorts having an Eastern Cooperative Oncology Group (ECOG) score of 0–1, and only a small number of subjects with ECOG 2 or higher (2, 26). However, one study did not report any details regarding the clinical status of the participants (14). One cohort reported a shorter OS in patients with a baseline ECOG performance status ≥ 1 compared to those with ECOG 0 (6.9 months vs. median not reached; p = 0.032) (22). Smoking status was analysed for nearly all patients, except those from one cohort (14). In each remaining papers, over 70% of patients were classified as current or former smokers, with one study enrolling a patient with unknown smoking status (27). However, patients without a history of smoking demonstrated significantly lower baseline sPD-L1 concentrations compared to smokers before receiving anti–PD-1 antibody treatment (n=10/39, p = 0.0062) (28). In the other studies, no significant associations were reported between serum or plasma sPD-L1 levels and clinical factors, including age, performance status or smoking history, across the original papers included in this review.

#### 3.2.3 Histological characteristics

The predominant histologic subtypes were adenocarcinoma and squamous cell carcinoma, with four studies also reporting cases of large cell carcinoma or clear cell carcinoma (14, 24, 25, 29). Although mutational status was not reported in three studies (22, 27, 28), and one cohort explicitly excluded patients with epidermal growth factor receptor (EGFR) mutations or anaplastic lymphoma kinase (ALK) rearrangements (14), individuals with EGFR or ALK alterations were still included in the remaining six study populations. Furthermore, a ROS1 fusion was identified in one patient (24), while HER2 and BRAF mutations were reported in four patients (29). Tissue PD-L1 expression was assessed in almost all studies, except for one that did not report any information regarding this biomarker (24). Most patients demonstrated positive PD-L1 expression, with ≥1% presence on tumor cells. However, two cohorts did not report tissue PD-L1 status for a subset of participants (22, 23). Importantly, increasing tumor PD-L1 TPS was associated with higher serum sPD-L1 levels (<1%: 64.4 ± 17.5 pg/mL; 1–49%: 70.6 ± 18.0 pg/mL; ≥50%: 77.7 ± 28.9 pg/mL; p = 0.0101), with a weak but significant correlation between serum and tumor PD-L1 expression (r = 0.214, p = 0.001) in one study (2). In another study, baseline functional sPD-L1 levels differed significantly between patients with high versus low tissue PD-L1 expression (p = 0.0162) (28). Except for one cohort showing higher sPD-L1 levels in squamous cell carcinoma than in adenocarcinoma, and two studies reporting associations between soluble immune checkpoint levels and tumor PD-L1 expression, no other included study found significant correlations between histologic subtype, mutational status, or tissue PD-L1 and circulating sPD-L1 levels. (2, 22, 28).

Most studies enrolled patients with advanced or recurrent NSCLC, ranging from stage IIIB to stage IV, with three cohorts also including stage II patients (24, 27, 28). Three original papers did not report disease stage classification (22, 23, 25). Thus, most NSCLC included patients treated with immunotherapy in these studies had metastatic disease, with up to 97% of subjects being stage IV in one of the papers (26). Although most of the included patients were characterized by metastatic disease, only three studies classified patients according to the metastatic sites (e.g., brain, liver, lung) (2, 25, 26), while one study categorized them based on the number of involved sites (M1a, M1b, M1c) (24). Higher serum levels of sPD-L1 were observed in patients with liver metastasis compared to those without (80.9 ± 36.6 pg/mL vs. 70.0 ± 21.8 pg/mL, p = 0.015) (2). Moreover, one cohort reported a significant association between elevated serum sPD-L1 levels and both liver (p = 0.041) and brain metastases (p = 0.03) (26).

#### 3.2.4 Treatment management

The treatment landscape consisted of anti-PD-1 antibodies (Pembrolizumab or Nivolumab, the latter investigated in all ten studies) and anti-PD-L1 antibodies (Atezolizumab, Durvalumab), administered either as monotherapy or in combination with chemotherapy. However, one study included a patient who received a combination of Nivolumab and the anti-CTLA-4 antibody, Ipilimumab (22). The majority of patients received immunotherapy as second-, third-, or later-line treatment, with three studies not enrolling any subjects in the first-line setting (24, 25, 29). However, two cohorts primarily consisted of patients receiving first-line treatment, with over 50% of subjects being treatment-naive (14, 27). Moreover, one study demonstrated that patients receiving first-line immunotherapy had higher levels of sPD-L1 compared to those treated in the second or third line, with this difference reaching statistical significance (p = 0.034) (27).

Except for one study, the included papers consistently reported that treatment response was associated with sPD-L1 levels (23). The overall response rate (ORR), comprising complete response (CR), partial response (PR), or stable disease (SD), varied across studies. While some papers did not observe any association between sPD-L1 levels and ORR (22, 27), and one cohort reported a non-significant trend toward higher sPD-L1 levels in non-responders (p = 0.379) (14), the majority of studies indicated that CR, PR, or SD were more frequently observed in patients with low circulating sPD-L1 levels compared to those with high levels (p = 0.0069) (24). The ORR in the high baseline sPD-L1 group was lower than in the low baseline sPD-L1 group (37% [95% CI: 22–53] *vs.* 57% [95% CI: 50–64]; p = 0.0158) (2). This trend was also observed dynamically after two months of treatment with Nivolumab, with an ORR of 90% in the low plasma sPD-L1 group (n = 10) compared to only 30% in the high plasma sPD-L1 group (n = 23; p = 0.002) (29). Moreover, progressive disease (PD) was more frequently observed in patients with high baseline plasma sPD-L1 levels compared to those with low levels (75% vs. 25%) (24).

Taken together, ten original papers included in the review reported the PFS and/or OS of 858 NSCLC cases treated with immune checkpoint inhibitors, according to sPDL1 cut-off values. Higher levels of sPD-L1 at baseline were associated in all studies included with poor progression free survival and reduced overall survival.

As previously mentioned, three original research papers included in the review enrolled both patients with NSCLC and individuals diagnosed with other malignancies, such as uveal melanoma, renal cell carcinoma, and head and neck squamous cell carcinoma (15), as well as gastric and bladder cancer (16) or melanoma (17), with lung cancer patients representing the majority in each cohort. In one cohort, no significant correlation was found between sPD-L1 levels and age, gender, cancer type, pathological status, or tissue PD-L1 expression (p = 0.5645) (16) while the other two studies did not report any such associations. Interestingly, in one study, NSCLC patients (n = 50; 39.1%) who showed a greater than 100% increase in sPD-L1 levels following ICI therapy had significantly longer progression-free survival (PFS) compared to those without such an increase. The median PFS was 6.3 months [95% CI: 0.0–19.4] versus 1.2 months [95% CI: 0.6–1.8], respectively (p = 0.029; log-rank test). Similarly, patients who experienced a ≥100% increase in sPD-L1 levels after ICI therapy demonstrated a significantly longer OS of 14.3 months [95% CI: 0.0–30.8], compared to 5.7 months [95% CI: 0.0–11.4] in those with a ΔsPD-L1 < 100% (p = 0.022; log-rank test) (17). The increase in sPD-L1 levels following ICI therapy may reflect effective tumor cell killing and the accompanying immune response. However, because this finding has not been consistently reproduced across studies (17), additional longitudinal studies evaluating the dynamic changes in sPD-L1 during treatment are needed to clarify its biological and prognostic significance.

### 3.3 Perioperative sPD-L1 levels in resectable NSCLC are associated with relapse free survival and OS

Three original papers included NSCLC patients who subsequently underwent surgical intervention, with determination of perioperative plasma levels of sPD-L1 (9, 21) or only postoperative quantification (20) (**Table 1**). While one study also included patients, who had received neoadjuvant (10.8%) or adjuvant (47.2%) treatment, the other two studies enrolled only individuals naive to any oncological treatment. Patients included were staged IA–IIIB, with one paper also analyzing stage IV patients (n = 2, 3.2%) (21). Regarding the clinicopathological characteristics, two papers reported no statistically significant differences in plasma sPD-L1 levels (9, 20). However, one study described higher plasma sPD-L1 levels in males (65.7 pg/mL vs. 42.6 pg/mL, p *=* 0.025) and in smokers (68.3 pg/mL vs. 47.2 pg/mL, p *=* 0.032). Higher plasma sPD-L1 levels before surgical intervention were associated with disease recurrence in one cohort (n = 74; recurrence vs. no recurrence: 966.44 pg/mL [95% CI: 576.53–1322.97] vs. 448.24 pg/mL [95% CI: 271.06–756.18]; p *=* 0.038), but this finding could not be validated in two other smaller cohorts (n = 43; n = 30) (9). Two studies showed higher sPD-L1 levels after surgical intervention compared with preoperative levels (from 63.4 pg/mL to 72.2 pg/mL at one month after surgery, p *<* 0.001) (21) and measured at a median of 20 days post-surgery (range 8–90) was 834.5 pg/mL (583.0–1548) versus 496.1 pg/mL (271.1–919.8), p *=* 0.033 (9). Nevertheless, when analyzed at three months or later (median 112 days; 95% CI: 105–189 (9);), plasma sPD-L1 levels were comparable to baseline values (62.0 pg/mL, p *=* 0.019 (21). Finally, one paper compared plasma sPD-L1 levels in NSCLC patients with those in healthy subjects, reporting statistically significant differences (p *<* 0.001) (20).

**Table 1:** Overview on HR estimates and 95% CIs for PFS and OS according to cut-offs used to classify patients by low or high baseline soluble immune checkpoint levels.

Study	n (H, L)	PFS						OS					Sample type
		Cut-off	HR (CI)	High	Low	*p*-value	Cut-off		HR (CI)	High	Low	*p*-value	
sPD-L1													
Zhao et al. (17)	126 (H = 52, L = 74)	–	–	–	–	–	89.6 pg/mL		1.611 (0.787-2.830)	18.8 months (13.8-23.9)	29.0 months (18.8-39.1)	0.179	plasma
	86 (H = 44, L = 42)	–	–	–	–	–	96.5 pg/mL		1.689 (1.009–2.827)‡	15.5 months (8.2-22.9)	27.8 months (19.7-36)	0.005	plasma
Okuma et al. (19)	96 (H = 40, L = 56)	–	–	–	–	–	7.32 ng/mL		–	13 months	20.4 months	0.037	plasma
Okuma et al. (24)	39 (H = 15, L = 24)	3.357 ng/mL	–	1.48 months	5.36 months	0.032	3.357 ng/mL		2.703 (1.041-7.692)*	7.2 months	NR/15 months	0.04	plasma
Costantini et al. (29)	43 (H = 24, L = 9)	33.97 pg/mL	4.85 (1.02-NR)	2.2 months (1.6-9.1)	11.8 months (6.5-NR)	0.008	33.97 pg/mL		3.225 (1.000- 5.667)‡	6.2 months (2.4-NR)	NR/20 months (13.6-NR)	0.028	plasma
Castello et al. (22)	20 (H = 10, L = 10)	27.22 pg/mL	–	5.8 months	4 months	0.595	27.22 pg/mL		–	18.8 months	8.1 months	0.483	plasma
He et al. (20)	88 (H = 66, L = 22)	–	–	–	–	–	3.4 ng/mL		0.372 (0.162–0.854)	61.73 months (55.9–67.55)	45.87 months (33.97-57.77)	0.02	plasma
Murakami et al. (2)	233 (H = 41, L = 192)	90 pg/mL	1.677 (1.119–2.512)	57 days (0-130)	177 days (126-218)	0.012	90 pg/mL		2.663 (1.671–4.245)	182 days (40-323) **6 months (1.3-10.7)	NR/1200 days **40 months	< 0.001	serum
Mazzaschi et al. (26)	109	113 pg/mL	2.55 (1.50-4.32)	3.8 months	11.9 months	<0.001	113 pg/mL		2.53 (1.42-4.51)	5.8 months	15.0 months	0.001	serum
Tiako Meyo et al. (30)	51 (H = 27, L = 24)	0.057 ng/mL	2.68 (1.36-5.28)	–	–	0.004	0.057 ng/mL		2.68 (1.23-5.84)	–	–	0.013	plasma
Oh et al. (17)	128 (H = 64, L = 64)	11 pg/μL	1.928 (1.038–3.581)	2.9 months (2.1-3.7)	6.3 months (3-9.6)	0.023	11 pg/μL		1.788 (1.207–2.650)	7.4 months (6.3-8.5)	13.3 months (9.2-17.4)	0.005	serum
Himuro et al. (23)	120 (H = 66, L = 54)	55.3 pg/ml	15.4 (1.10–86.7)	76 days **2.5 months	132 days **4.3 months	0.019	–		–	–	–	–	plasma
	122 (H = 19, L = 103)	–	–	–	–	–	92.9 pg/ml		11.4 (1.19-52.3)	115 days **3.8 months	444 days **14.8 months	< 0.001	plasma
	90 (H = 48, L = 42)	63.7 pg/ml	6.09 (1.42-21.0)	64 days **2.1 months	239 days **7.9 months	<0.001	–		–	–	–	–	plasma
	92 (H = 19, L = 73)	–	–	–	–	–	99.8 pg/ml		42.6 (6.83-226)	118 days **3.9 months	653 days **21.8 months	<0.001	plasma
Chmielewska et al. (14)	120 (H = 59, L = 61)	20 pg/mL	1.661 (1.101–2.506)*	2.5 months (2-4)	5.8 months (5-8.5)	0.0156	20 pg/mL		1.867 (1.185–2.942)*	7 months (4-13.5)	16.5 months (12-23.8)	0.0071	plasma
Hayashi et al. (25)	50 (H = 29, L = 21)	205 pg/ml	2.86 (1.47-5.56)*	2.2 months	9.1 months	0.002	–		–	–	–	–	plasma
F.O.Milder et al. (9)	74 (H = 24, L = 50)	1000 pg/mL	3.349 (1.728- 6.492)	15.5 months	45.0 months	<0.001	1000 pg/mL		4.343 (1.646–11.461)	33.0 months	48.0 months	0.003	plasma
Han et al. (6)	70 (H = 36, L = 34)	–	–	–	–	–	713.75 pg/mL		1.858 (1.011–3.416)	197 days **6.4 months	643 days **21.8 months	0.0277	plasma
sPD-L2													
Costantini et al. (29)	43	–	–	–	–	–	–		–	–	–	NS	plasma
Han et al. (6)	70 (H = 35, L = 35)	3233.18 pg/ml	–	–	–	–	–		1.060 (0.579–1.942)	–	–	NS	plasma
sPD-1													
Himuro et al. (23)	90	205.1 pg/mL	1.68 (0.41–5.30)	–	–	0.426	–		1.38 (0.41-5.30)	–	–	0.67	plasma
	90 (H = 67, L = 23)	5801 pg/ml	–	165 days	62 days	0.002	–		–	–	–	–	plasma
	90 (H = 68, L = 24)	5827 pg/ml	–	–	–	–	–		–	821 days	183 days	0.001	plasma
Hayashi et al. (25)	50 (H = 26, L = 24)	135 pg/mL	0.78 (0.41–1.50)	2.8 months	5.2 months	0.459	–		–	–	–	–	plasma
Tiako Meyo et al. (30)	51 (H = 15, L = 36)	0.156 ng/ml	2.59 (1.29–5.21)	–	–	0.007	–		2.28 (1.11-4.68)	–	–	0.025	plasma
sCTLA-4													
Hayashi et al. (25)	50 (H = 21, L = 29)	1.85 pg/mL	0.54 (0.27–1.06)	5.7 months	2.7 months	0.074	–		–	–	–	–	plasma

*Marks the data for which the original HR was reported as High over Low; ** marks the computed month numbers starting form original days number data; ‡ marks the computed HR of OS data analyzed from the high and low original values.

### 3.4 Other soluble immune checkpoints and their association with survival

PD-L2, a key ligand of PD-1, has an unclear clinical role, while soluble PD-L2 has been suggested as a prognostic biomarker in multiple cancers, including NSCLC, melanoma, and ovarian cancer (13). Although several studies have reported sPD-L2 as a potential biomarker for NSCLC alongside sPD-L1, no significant associations with PFS or OS were observed. Moreover, even when patients were divided into two groups (high sPD-L2 vs. low sPD-L2) using a cutoff value of 3233.18 pg/mL, with equal numbers in each subgroup (n = 35), no statistically significant difference in overall survival was found (6).

One of the most extensively studied co-inhibitory immune checkpoint receptors is PD-1, which interacts with its ligands PD-L1 and PD-L2. Through mechanisms such as alternative splicing, PD-1 can generate soluble isoforms that have been proposed as predictive biomarkers in cancer screening. While some studies did not demonstrate a statistically significant association between pre-treatment plasma sPD-1 and PFS or OS in patients treated with immunotherapy combined with chemotherapy [p = 0.426 for PFS; p = 0.670 for OS, (23)] or ICI monotherapy [p = 0.228 and p = 0.729, (23); p = 0.459, (25)], other studies reported a strong correlation between post-treatment sPD-1 and PFS/OS in patients receiving anti-PD-1 antibodies (Nivolumab or Pembrolizumab). A significant association was observed for both PFS (p = 0.002) and OS (p < 0.001) (23). Therefore, higher post-treatment sPD-1 levels are associated with improved survival (23). Moreover, one study reported that the sPD-L1/sPD-1 ratio significantly influenced patient survival (p < 0.05). Using the median value of 1.7925 as the cutoff, patients with a ratio ≤ 1.7925 had a mean OS of 54.44 months [95% CI: 46.37–62.51], compared to 65.10 months for those with a ratio > 1.7925 [95% CI: 58.39–71.81], with survival rates of 51.2% and 72.7%, respectively (20). Additionally, another cohort established a composite criterion (sCombo), defined as positivity for sPD-1 and/or sPD-L1 in each patient. In univariate analysis, baseline sCombo positivity was associated with a significant reduction in PFS (78 days [55–109] vs. 658 days [222–not reached]; HR 4.12, 95% CI: 1.95–8.71; p = 0.0002) (30).

sCTLA-4, a splice variant of CTLA-4, suppresses immune responses and has been linked to improved outcomes with ipilimumab therapy (31). Circulating sCTLA-4 levels are low and difficult to measure with standard ELISA kits, which has hindered research on its clinical utility. Pre-treatment sCTLA-4 shows only a trend toward association with PFS (p = 0.074) (25).

### 3.5 Lower circulating sPD-L1 levels are associated with improved clinical outcomes: progression-free survival and overall survival

**Figure 2:**
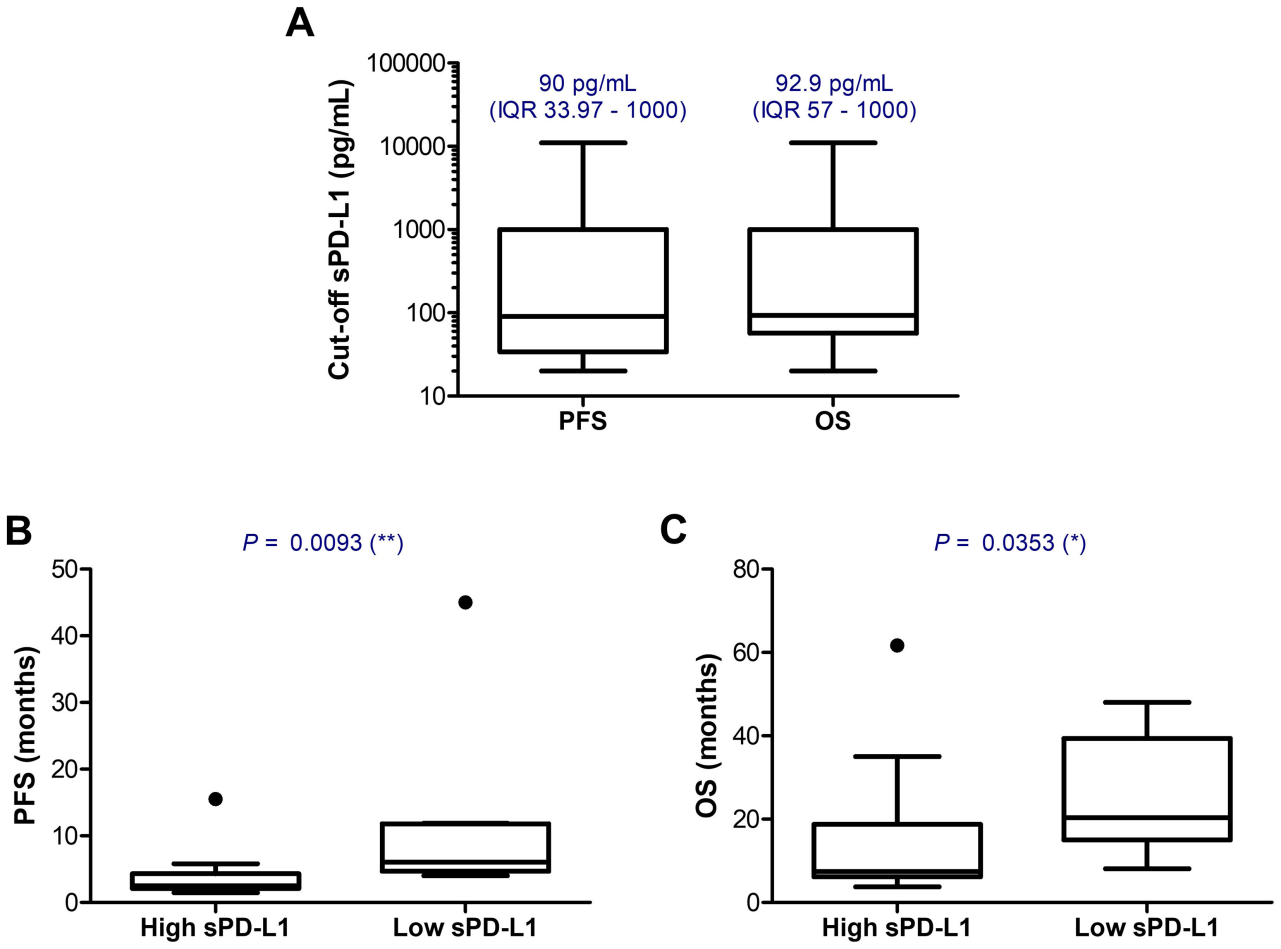
Soluble PD-L1 (sPD-L1) levels in relation to progression-free survival (PFS) and overall survival (OS) in NSCLC patients. **(A)** Cut-off sPD-L1 values used for evaluating PFS or OS differences between the low and high baseline sPD-L1 groups. **(B)** PFS (in months) stratified by high versus low baseline sPD-L1 levels (**p < 0.01, Wilcoxon signed-rank test). **(C)** OS (in months) stratified by high versus low baseline sPD-L1 levels (*p < 0.05, Wilcoxon signed-rank test).

In these analysed studies, patients were stratified into high and low sPD-L1 groups using predefined cut-off values (**Table 1**). The distribution of serum cut-off sPD-L1 values (pg/mL) in relation to both PFS and OS showed median levels of 90 pg/mL (IQR 33.97–1000) for the PFS cohort and 92.9 pg/mL (IQR 57–1000) for the OS cohort, indicating a largely similar pattern across both survival endpoints (**Figure 2A**). Importantly, patients with high sPD-L1 levels had significantly shorter PFS (2.5 months, 95% CI: 2.11-4.3) compared with those in the low sPD-L1 group (6.05 months, 95% CI: 4.68-11.83; p = 0.0093), indicating that elevated circulating sPD-L1 is associated with more rapid disease progression (**Figure 2B**). Similarly, OS was significantly lower in patients with high sPD-L1 levels (p = 0.0353), suggesting that sPD-L1 may serve as a prognostic marker, reflecting a more aggressive tumor phenotype or a systemic immunosuppressive environment (**Figure 2C**). Overall, these results demonstrate that elevated baseline circulating sPD-L1 levels are consistently associated with shorter progression-free and overall survival. The comparable distributions suggest that this prognostic effect is not driven by extreme values, supporting the potential of sPD-L1 as a non-invasive biomarker to identify patients at higher risk of disease progression and reduced survival.

**Figure 3:**
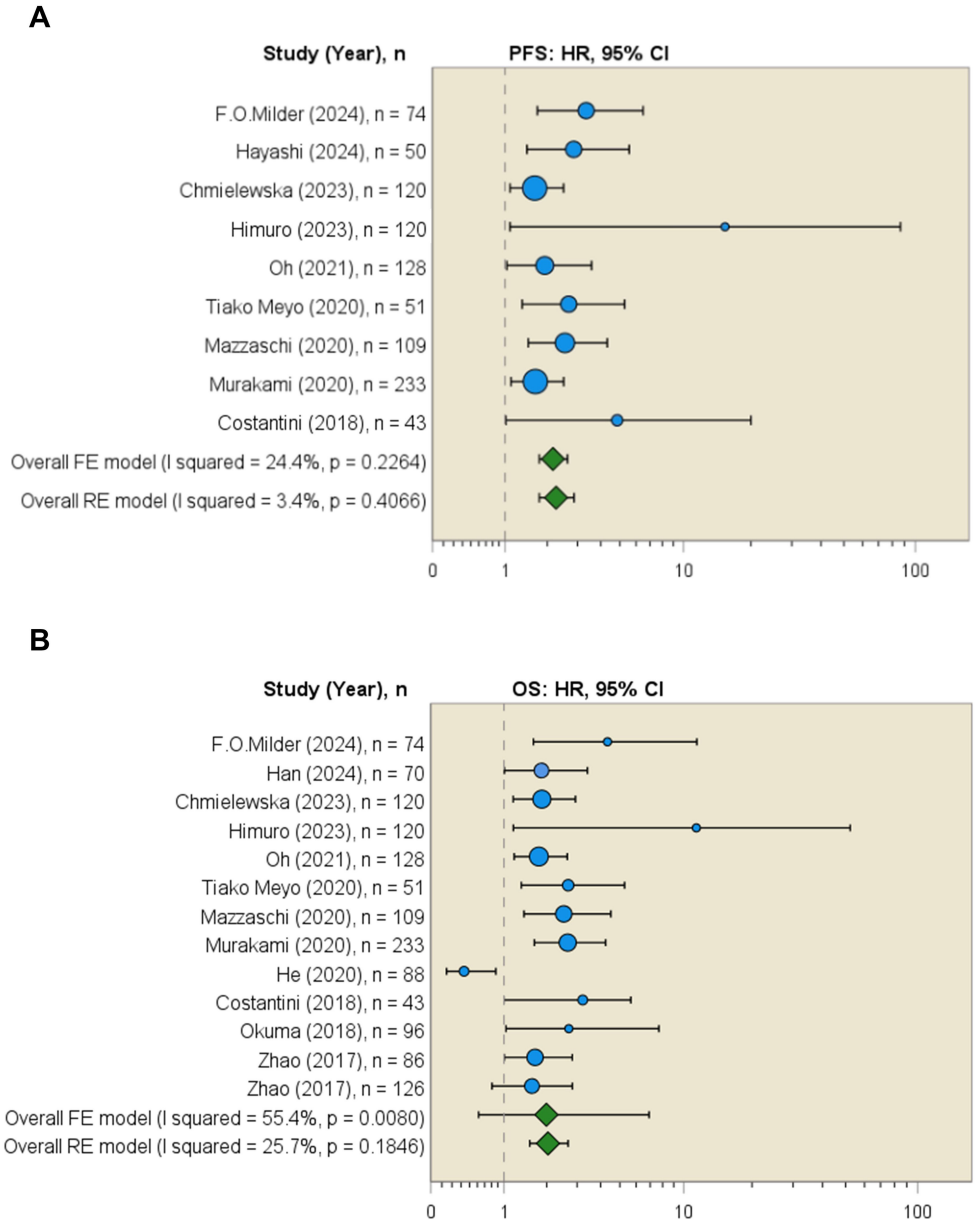
Forest plots for **(A)** progression-free survival and **(B)** overall survival. The forest plots display the effect sizes (HR) and 95% confidence intervals (CI) for each study included in the individual analysis, sorted by publication date. Each horizontal line represents an individual study’s effect size and CI, with the study’s weight indicated by the size of the blue circle. The green diamond at the bottom represents the overall HR estimate and 95% CI, calculated using both fixed-effect and random-effects models.

The forest plot analysis of PFS indicates that elevated sPD-L1 levels are associated with a significantly higher risk of disease progression in NSCLC (**Figure 3A**). Across the nine included studies published between 2018–2024, the majority reported hazard ratios (HR) above 1, demonstrating a consistent adverse effect. Precision varied by study size, with smaller studies (Constantini, n = 43; Tiako Meyo, n =51) showing wide confidence intervals, while larger studies (Murakami, n=233; Chmielewska, n = 120; Oh, n = 128) yielded narrower, more precise estimates. Two studies (23, 29) exhibited considerable uncertainty, likely due to small sample sizes or low event rates, and therefore had limited influence on the pooled effect. Between-study heterogeneity was low in both fixed-effect (FE) and random-effect (RE) models (FE model: I² = 24.4%, p = 0.2264; RE model: I² = 3.4%, p = 0.4066), suggesting that the effect is largely consistent across studies. The pooled estimates, represented by the diamonds in the forest plot, were above 2 and their confidence intervals did not cross 1, supporting a statistically significant and robust association between higher sPD-L1 levels and shorter PFS (FE model: pooled HR 2.16, 95% CI: 1.77-2.64, p < 0.0001; RE model: pooled HR 2.26, 95% CI: 1.77-2.87, p < 0.0001). When applying the random-effect model to investigate the role of sPD-1 levels based on the 3 studies reporting data on PFS (detailed in **Table 1**), the pooled HR reached a non-significant value of 1.34 [95% CI: 0.83-2.16, p = 0.2355], while the cut-off value ranged from 135 pg/mL to 205.1 pg/mL.

The second forest plot presents a pooled analysis of overall survival (OS) across the studies included evaluating the prognostic impact of a specific biomarker or intervention (**Figure 3B**). Most studies show HRs at or above 1, indicating a trend toward poorer survival, though some, such as Chmielewska (2023) and Tiako Meyo (2020), have wide confidence intervals reflecting smaller sample sizes or study variability. The pooled estimates, shown as green diamonds, indicate an overall significant HR above 2 under the RE model (pooled HR 2.04, 95% CI 1.55-2.68, p < 0.0001). Heterogeneity was moderate in the FE model (I² = 55.4%, p = 0.0080) but lower in the RE model (I² = 25.7%, p = 0.1846), suggesting some variability between studies. Despite this, the consistency of point estimates above 1 supports a robust association between the evaluated factor and reduced OS, while highlighting the need to consider study differences.

If, for soluble PD-L1, the overall effect showed a significant increase in PFS and OS among patients with baseline low levels of sPD-L1, the results for soluble PD-1 (sPD-1) were inconsistent. More precisely, when estimating PFS, the random-effects model based on the three studies on sPD-1 detailed in **Table 1** indicated a non-significant difference (pooled HR 1.34 95% CI 0.83-2.16, p = 0.2355), confirmed by moderate-to-high heterogeneity (I² = 62, 24%, p = 0.0708).

### 3.6 Publication bias

**Figure 4:**
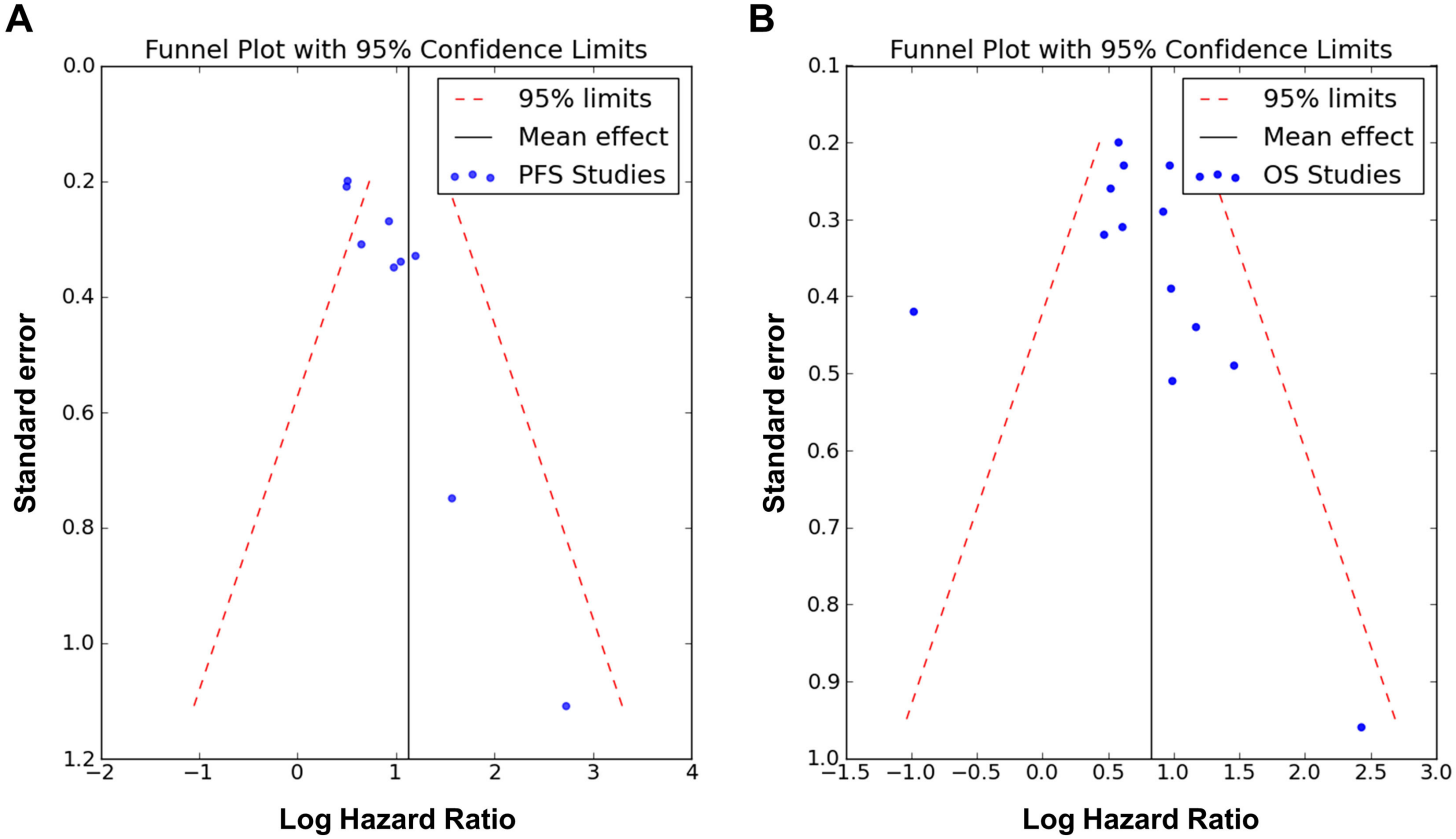
Funnel plots for **(A)** progression-free survival and **(B)** overall survival. The funnel plots display the effect sizes (logHR) and the corresponding standard error (SE of logHR) for each study included in the individual analysis.

**Figure 5:**
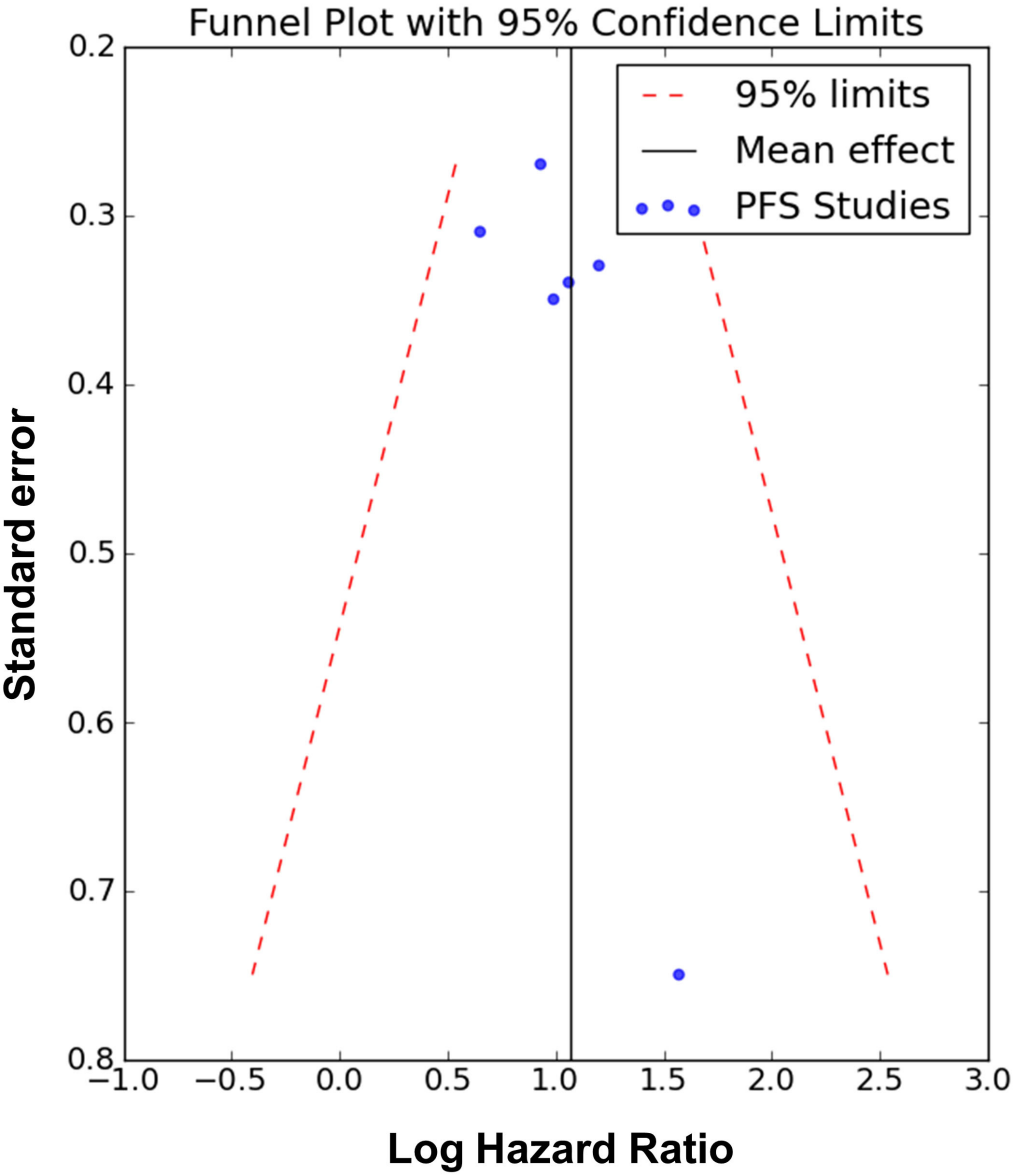
Funnel plots for progression-free survival (PFS) of the improved model. The funnel plots display the effect sizes (logHR) and the corresponding standard error (SE of logHR) for each study included in the individual analysis.

To assess potential publication bias, we visually examined the funnel plots for both endpoints (PFS and OS) to evaluate asymmetry and performed Egger’s test. The visual inspection of the funnel plots suggested possible asymmetry for PFS, which was confirmed by Egger’s test (p = 0.0018, **Figure 4A**). For OS, Egger’s test yielded a p-value of 0.4227, indicating no significant asymmetry (**Figure 4B**). Consequently, we excluded the studies by Murakami (2020), Chmielewska (2023), and Himuro (2023) from the PFS analysis (**Figure 5**). After exclusion, Egger’s test produced a p-value of 0.2024, suggesting the absence of publication bias. When these studies were included in a separate meta-analysis, the pooled PFS hazard ratio (HR) reached 2.65 [95% CI: 2.02–3.49, p < 0.001] for both fixed-effects and random-effects models.

## 4 Discussions

Immune checkpoint inhibition has significantly transformed the treatment landscape for most solid tumors, particularly NSCLC. Anti-PD-1 or anti-PD-L1 antibodies have been incorporated into lung cancer management, either as monotherapy or in combination with chemotherapy, showing excellent results in terms of progression-free survival, and especially in overall survival and quality of life (6). Tissue PD-L1 expression assessed by immunohistochemistry remains the only validated biomarker currently used to guide treatment selection (32). Despite this, increased tissue PD-L1 expression does not reliably correlate with a successful response to immunotherapy, with only a limited subset of patients experiencing meaningful benefit (12, 32). Therefore, there is an unmet clinical need in pursuing biomarkers, to support clinical decision making, with regard to the selection of a treatment approach (e.g., chemotherapy alone, chemo-immunotherapy, or immunotherapy monotherapy) (33). Although the guidelines rely on tissue PD-L1 expression as a criteria for treatment selection, many patients experience progression, hyper-progression, or immune-related adverse effects (34, 35). All of these scenarios compromise quality of life and place a financial burden on the healthcare system. In addition, tissue PD-L1 expression cannot be assessed dynamically and does not capture the molecular changes occurring in the tumor microenvironment during disease progression (36, 37). Hence, NSCLC management requires relevant biomarkers that are easy to use in clinical practice and accessible through minimally invasive procedures. In this context, soluble PD-L1 levels and other soluble immune checkpoint molecules have been increasingly assessed over the past decade in serum or plasma, either at baseline or dynamically, potentially reflecting the biological behavior of lung tumor evolution (27, 38).

**Figure 6:**
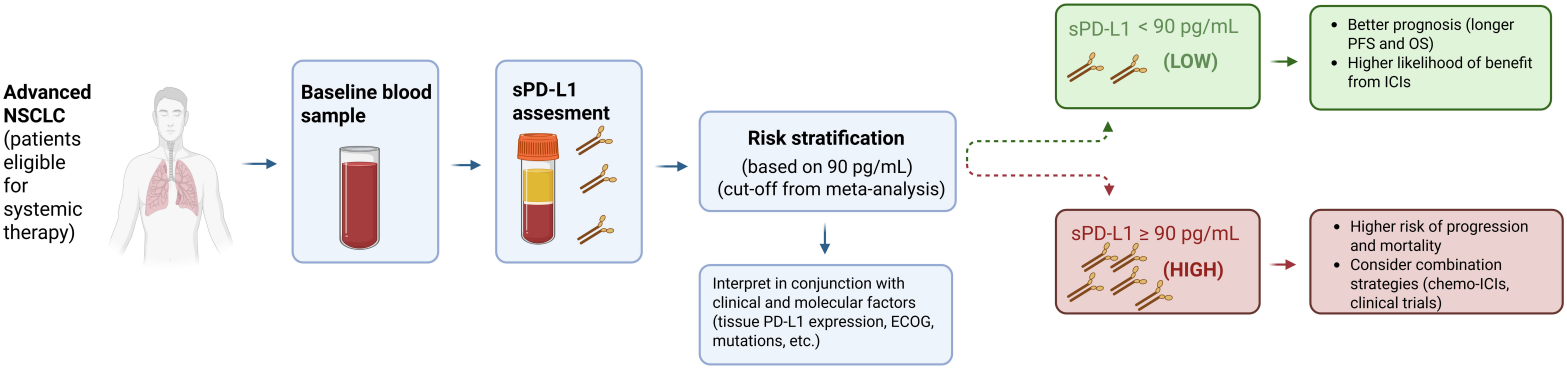
Patients with sPD-L1 levels below the 90 pg/mL threshold identified in this meta-analysis may have a better prognosis and greater likelihood of benefiting from ICIs. Elevated sPD1-L1 levels may indicate a higher-risk population requiring more intensive therapeutic strategies and follow-up. This approach remains to be validated in prospective studies.

**Figure 7:**
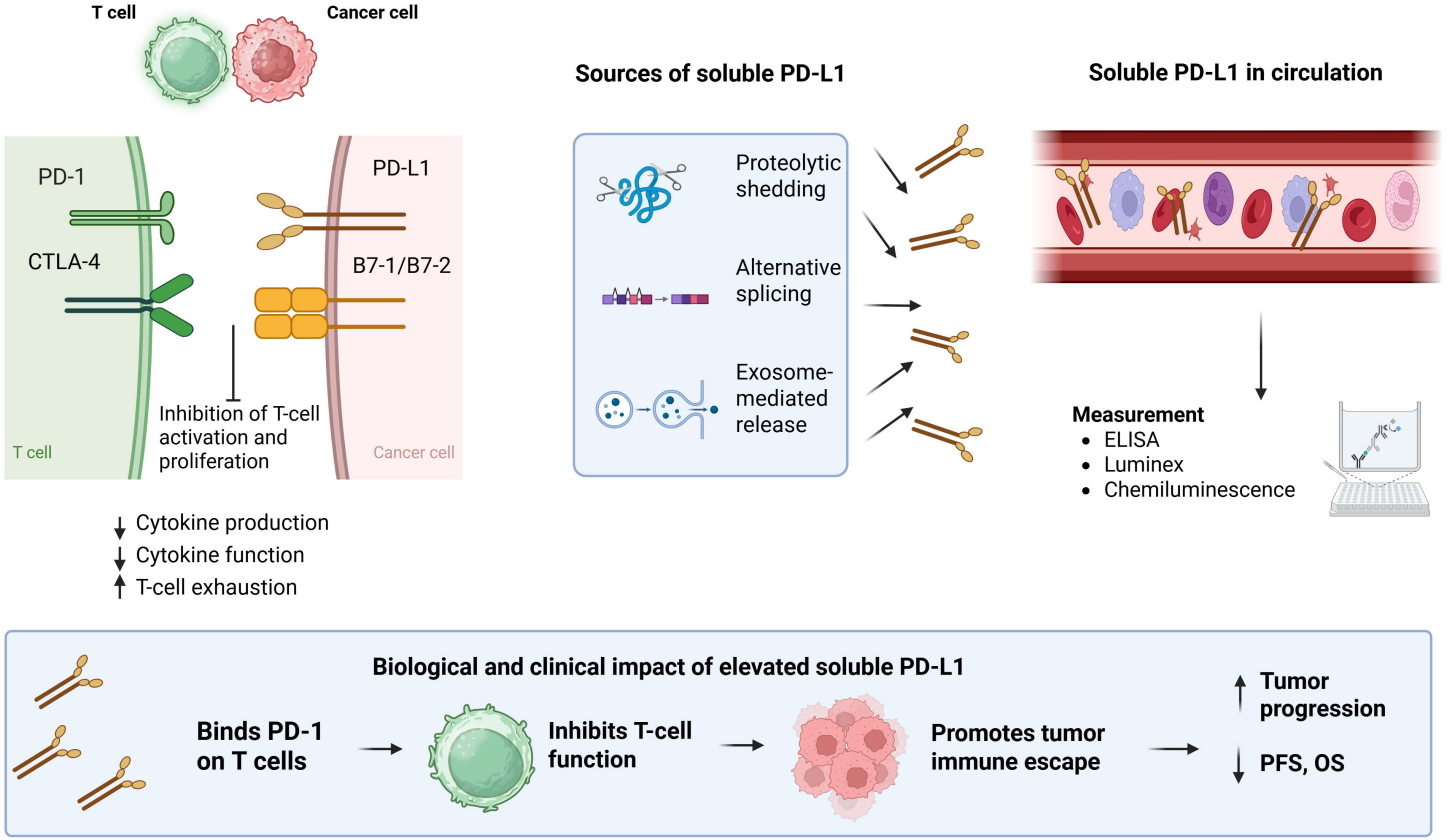
Schematic diagram of meta-analysis findings. Tumor- and immune cell–derived PD-L1 can enter the circulation through proteolytic shedding, alternative splicing, or exosome-mediated release. Circulating sPD-L1 remains biologically active, binds to PD-1 on T cells, and inhibits their activation and antitumor function. This mechanism promotes immune evasion and has been associated with adverse clinical outcomes in non–small cell lung cancer (NSCLC).

Several studies have investigated sPD-L1 levels in cancer patients; however, due to heterogeneity in study populations, the lack of standardized methods for measurement, and variability in reported findings, its role in clinical practice remains unclear. Therefore, it is essential to determine whether sPD-L1 is associated with clinical outcomes or could guide NSCLC management strategies (37, 39). Based on these observations, this systematic review aimed to investigate the prognostic value of soluble immune checkpoints (including sPD-L1, sPD-1, and sCTLA-4) in predicting progression-free survival (PFS) and overall survival (OS) in NSCLC patients. Additionally, the study sought to identify a potential baseline cut-off value for soluble PD-L1 for predicting PFS and OS, reporting median values and interquartile ranges (IQR): 90 pg/mL (IQR 33.97–1000) for PFS (**Figure 6**) and 92.9 pg/mL (IQR 57–1000) for OS. A subsequent meta-analysis revealed heterogeneity across studies; however, pooled hazard ratios (HRs) above 2 were observed for both PFS and OS in random-effects models, indicating a significant association between low baseline soluble PD-L1 levels and improved outcomes (**Figure 7**).

### 4.1 Association of low sPD-L1 with improved outcomes

Across most studies, elevated levels of sPD-L1 are associated with poor PFS and OS, while others failed to identify an association between high blood levels and clinical outcomes. This discrepancy can be explained by the heterogeneity of the cohorts included in the studies, concerning patient enrolment number and their characteristics, the treatment protocols, and the measurement assay of the soluble factor in serum/plasma. Even though the sPD-L1 thresholds varied, Murakami et al., Okuma et al., and Costantini et al. reported unfavorable PFS and OS in patients treated with anti-PD-1 antibodies (Nivolumab or Pembrolizumab) when serum/plasma levels were above the cut-off value (2, 24, 29). Although the studies were able to find a significant association between the endpoints and the soluble factors, the authors did not demonstrate a correlation between fluctuations in serum/plasma sPD-L1 levels and survival outcomes (22). Mazzaschi et al., demonstrated a statistically significant association between high level of serum sPD-L1 and poor outcomes, in patients treated with both anti-PD-1 and anti-PD-L1 antibodies (Nivolumab, Pembrolizumab and Atezolizumab), through Cox uni- and mutivariate proportional hazards regression models (26). However, Costantini and colleagues, despite analysing sPD-L1 levels at baseline and at the first tumor evaluation, did not find a statistically significant difference between responders and non-responders (29). However, even though Castello et al. observed an increase in sPD-L1 levels in lung cancer patients after 3–4 cycles of Nivolumab or Pembrolizumab, no differences in OS or PFS were found between those with high and low levels (22). This incongruence can be explained by the small number of patients (n = 20) and the limited follow-up period (10.3 months) (22). When it comes to other treatment modalities (chemotherapy, chemo-radiotherapy, targeted therapies), more authors were able to demonstrate a strong correlation between low sPD-L1 and improved survival (6, 18, 19).

Moreover, soluble PD-L1 could represent an important prognostic and predictive marker for relapse in early-stage lung cancer treated with lobectomy or pneumonectomy, as demonstrated by F.O. Milder and colleagues (9). However, one original study that enrolled patients undergoing surgery for NSCLC reported a discrepancy regarding the association between sPD-L1 levels and survival (20). Higher levels of sPD-L1 and high sPD-L1/sPD-1 ratios are significantly associated with longer OS (sPD-L1 plasma level: 61.73 months [95% CI: 55.9-67.55] versus 45.87 months [95% CI: 33.97-57.77, p = 0.02]. This may be explained due the demographic and clinical composition of the study population (81.8% male patients, 52.3% middle and 45.5% poorly differentiated tumors). Moreover, stage IIIA patients, who underwent tumor resection following sPD-L1 evaluation and were therefore disease-free, were the most represented in the cohort (20). It has been shown that subjects with advanced-stage tumors exhibited higher median sPD-L1 levels (49.77 pg/mL; IQR 29.10–100.60) compared to those with early-stage disease (I–II) (median: 17.88 pg/mL; IQR 6.87–44.88; p < 0.0001) (40).

Along sPD-L1, others soluble immune checkpoints were evaluated as predictive/prognostic biomarkers for NSCLC management. Post-treatment sPD-1 was statistically associated with both PFS and OS in a cohort of patients treated with Nivolumab or Pembrolizumab (23). Furthermore, NSCLC patients receiving erlotinib (EGFR-TKI) who showed an increase in sPD-1 during treatment experienced significantly longer PFS and OS compared to those with decreasing or undetectable sPD-1 levels. Median PFS was 6.7 months [95% CI: 0–28.7] versus 6.8 months [95% CI: 4.2–9.5; p = 0.015], and median OS was 26.2 months [95% CI: 2.6–49.7] versus 10.7 months [95% CI: 7.0–14.4; p = 0.018] (41).

Nonetheless, these original studies have several limitations that may significantly impact PFS and OS. Most patients enrolled had poor performance status in multiple cohorts, with ECOG 2 and 3 patients ranging from 9% (2) to more than half of the study population (51.7%) (23). Additionally, treated patients mostly had advanced-stage disease and received therapy in later lines, with only a few studies including individuals at first-line treatment (14, 27). All these clinical characteristics may influence prognosis, potentially leading to shorter PFS or OS, thereby limiting the predictive/prognostic value of sPD-L1 (42). Finally, the cohorts included a small number of patients, with the smallest comprising only 20 individuals making it difficult to achieve statistically significant results (22).

### 4.2 Association of sPD-L1 levels with clinicopathological characteristics

**Figure 8:**
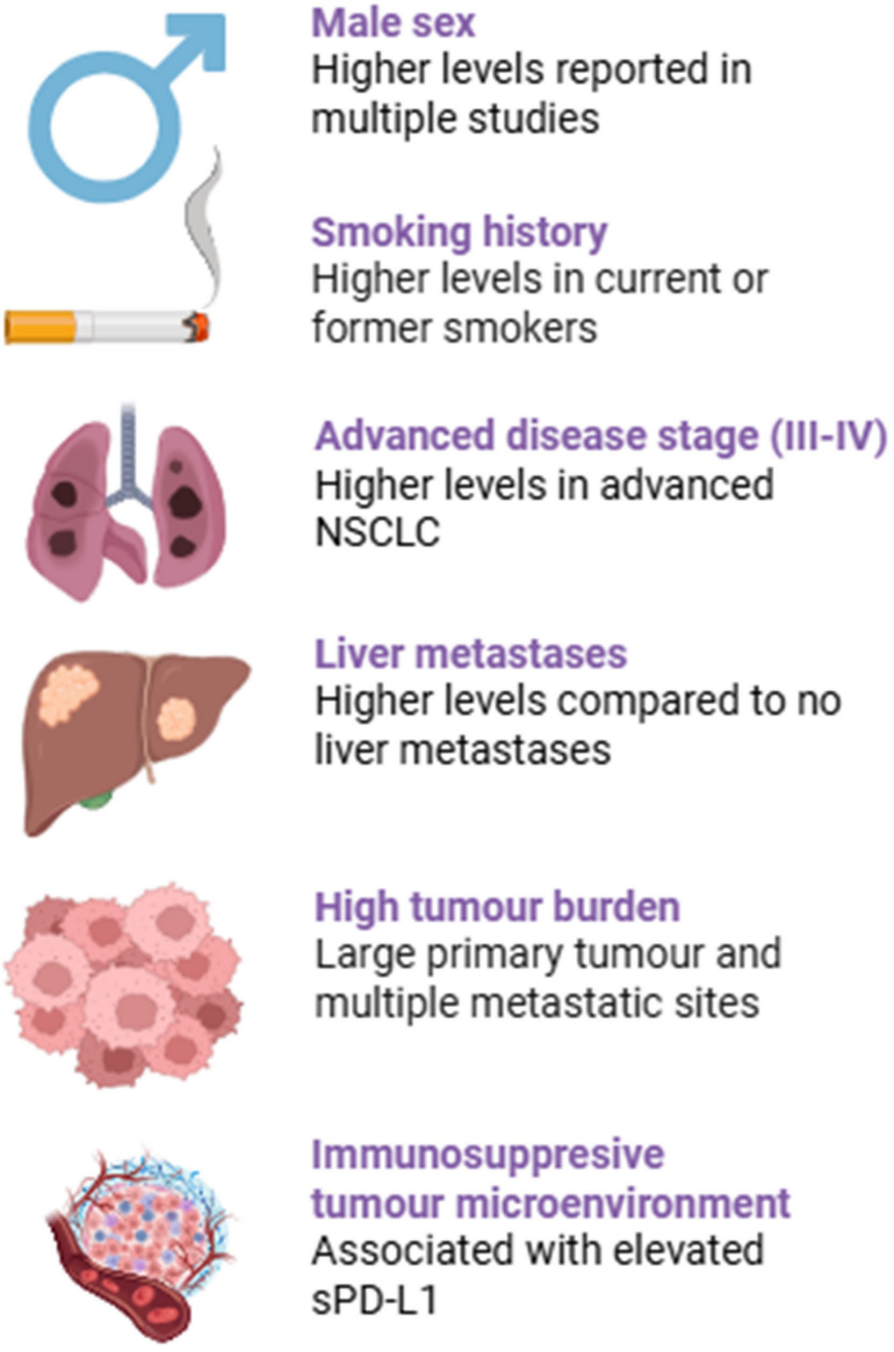
Factors associated with elevated baseline sPD-L1 levels in NSCLC. Higher sPD-L1 levels have been reported in male patients, smokers, advanced-stage disease, liver metastases, high tumor burden, and in an immunosuppressive tumor microenvironment.

**Figure 9:**
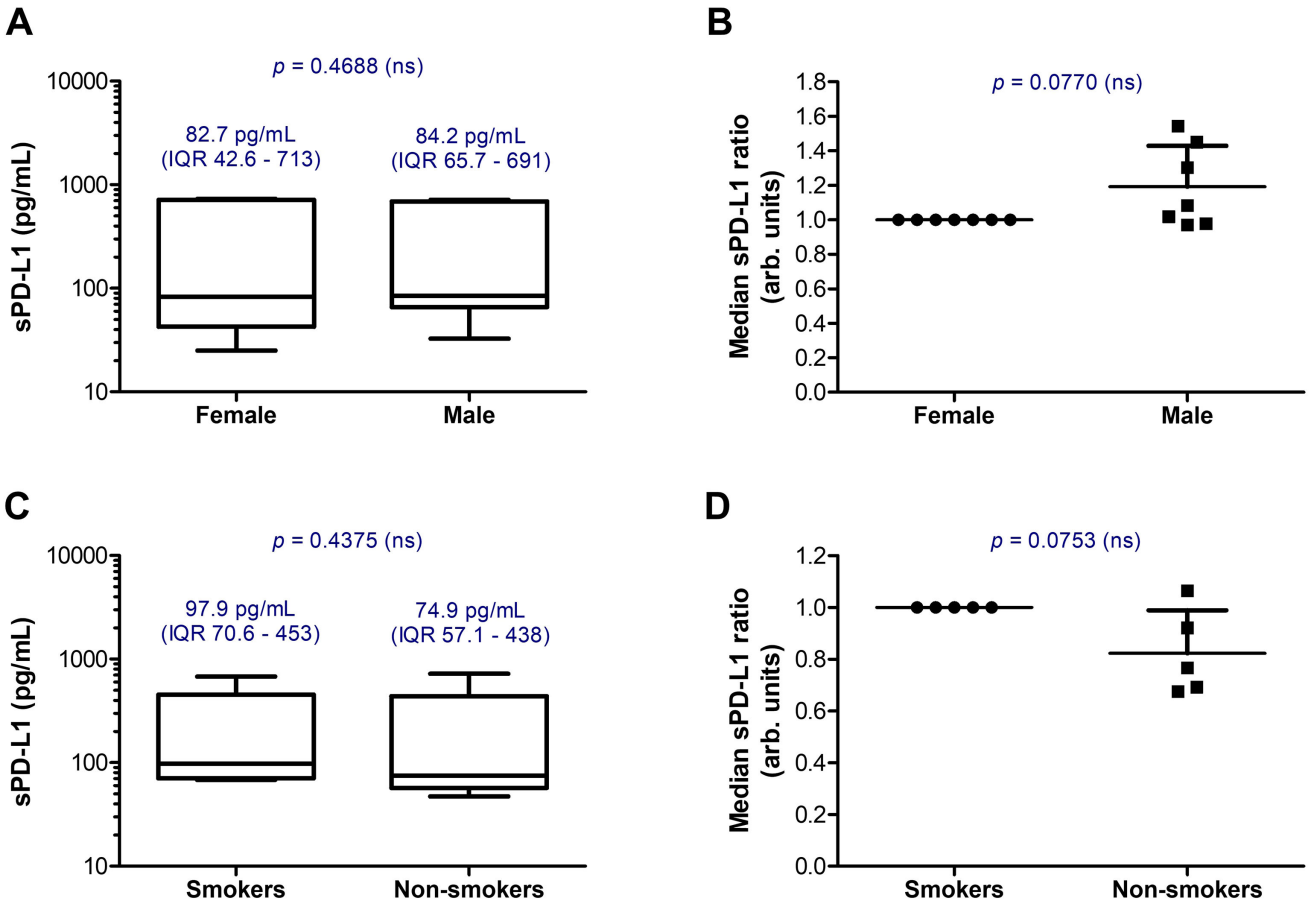
Soluble PD-L1 levels according to potential confounding factors: gender and smoking status. **(A)** sPD-L1 levels in female versus male patients (ns, not significant; Wilcoxon matched-pairs test). **(B)** Normalized male-to-female sPD-L1 ratio (ns, not significant; paired t-test). **(C)** sPD-L1 levels in smokers versus non-smokers (ns, not significant; Wilcoxon matched-pairs test). **(D)** Normalized non-smoker-to-smoker sPD-L1 ratio (ns, not significant; paired t-test).

Nevertheless, even if sPD-L1 were to become a standardized biomarker in clinical practice for NSCLC patients, it would be important to identify the patient profile for whom dynamic evaluation of this soluble immune checkpoint would be appropriate (**Figure 8**). For instance, several studies included in the review reported a tendency toward higher sPD-L1 values in males compared to females. Accordingly, Teramoto et al. demonstrated a statistically significant difference in preoperative plasma sPD-L1 levels, with higher values observed in male patients (median: 65.7 vs. 42.6 pg/mL, p = 0.025; **Table 2**) (21). However, pooled data from seven studies reporting median sPD-L1 levels in male and female patients did not reveal a statistically significant sex-related difference (**Figure 9A**). Nevertheless, the male-to-female ratio of the reported median sPD-L1 values was 1.19 ± 0.24, suggesting a tendency toward higher sPD-L1 levels in males, although this trend did not reach statistical significance (p = 0.077; **Figure 9B**). The limited number of studies and the heterogeneity among study populations and methodologies may have reduced the statistical power to detect significant differences. Despite these limitations, the available data suggest a possible trend toward lower sPD-L1 levels in female patients. Emerging evidence indicates that estrogen signaling may influence PD-L1 expression in NSCLC through several pathways, including ERα/ERβ signaling, EGFR activation, and the ERβ/SIRT1/FOXO3a axis. In line with this, high ERβ expression combined with increased aromatase has been associated with poorer survival in NSCLC. Although a direct link with circulating sPD-L1 has yet to be demonstrated, these findings suggest that hormonal signaling may contribute to the sex-related differences observed in soluble immune checkpoint levels (43, 44). Moreover, Castello et al. reported a shorter OS in female (4 vs 18 months, p = 0.016) (22).

**Table 2:** Overview of sPD-L1 levels in patients stratified according to confounding factors such as gender, smoking status, and histological subtype.

Study	Gender		Smoking status		Histological type		Sample type
	Female	Male	Smokers	Non-smokers	SCC	Non-SCC	
sPD-L1							
Okuma et al. (19)	7.13 ± 2.92 ng/mL [95% CI: 4.09–20.0] (n = 42)	6.91 ± 2.90 ng/mL [95% CI: 2.29–17.08] (n = 54)	6.79 ± 3.09 ng/mL [95% CI: 2.29–20.0] (n = 60)	7.22 ± 2.54 ng/mL [95% CI: 4.98–14.79] (n = 36)	5.95 ng/mL (n = 7)	ADC: 6.91 ± 2.73 ng/mL [95% CI: 2.29–17.08] (n = 73) Large cell carcinoma: 8.20 ± 2.70 ng/mL [95% CI: 6.29–13.8] (n = 1)	plasma
Okuma et al. (24)	1.58 ng/mL [95% CI: 0.26-5.15] (n =10)	2.29 ng/mL [95% CI: 1.41-4.33] (n = 29)	2.27 ng/mL [95% CI: 1.50-4.16] (n = 28 *heavy smokers)	1.53 ng/mL [95% CI: 0.22-4.32] (n = 11 *no/light, (Brinkmann index <400)	1.67 ng/mL [95% CI: 1.34-3.87] (n = 7)	2.27 ng/mL [95% CI: 0.56-4.38] (n = 32)	plasma
Castello et al. (22)	24.98 pg/mL (n = 7)	32.55 pg/mL (n = 13)	–	–	45.28 pg/mL	25.68 pg/mL	plasma
Murakami et al. (2)	68.0 ± 22.6 pg/mL (n = 81)	73.6 ± 25.9 pg/mL (n = 152)	72.8 ± 25.4 pg/mL (n = 179)	67.0 ± 21.8 pg/mL (n = 54)	72.4 ± 23.3 pg/mL (n = 52)	71.5 ± 25.4 pg/mL (n = 181)	serum
Mazzaschi et al. (26)	82.7 ± 48.6 pg/mL (n =36)	84.2 ± 52.3 pg/mL (n = 73)	Current: 97.7 ± 54.1 pg/mL (n = 36) Ex-smoker:78.8 ± 51.1 pg/mL (n = 48)	74.9 ± 33.9 pg/mL (n = 25)	77.8 ± 31.7 pg/mL (n = 32)	85.8 ± 31.2 pg/mL (n = 7)	serum
Teramoto et al. (21)	42.6 pg/mL (n = 18)	65.7 pg/mL (n = 45)	68.3 pg/mL (n = 47)	47.2 pg/mL (n = 16)	58.9 pg/mL (n = 17)	65.5 pg/mL (ADC) (n = 43)	
Han et al. (6)	730.5 pg/mL [95% CI: 318.4-3656] (n = 34)	713.8 pg/mL [95%CI: 240.6-3815] (n = 61)	–	–	768.9 pg/mL [95% CI: 348.2-1739] (n =19)	ADC: 681.9 pg/mL [95% CI: 251.6-3815] (n = 61); SCLC: 745.8 pg/mL[95%CI: 240.6-1065] (n = 10); Others: 568.0 pg/mL [95% CI: 380.0-3656] (n = 5)	plasma

SCC, Squamous Cell Carcinoma; ADC, Adenocarcinoma; CI, Confidence Interval.

Likewise, a general trend toward higher values in smokers than non-smokers was reported by Teramoto et al. (68.3 *vs.* 47.2 pg/mL, p = 0.032) (22). However, pooled data from the five available studies did not demonstrate a statistically significant difference between the two groups (**Figure 9C**). The median non-smoker-to-smoker sPD-L1 ratio was 0.828 ± 0.166 (p = 0.0753; **Figure 9D**), indicating a tendency toward higher sPD-L1 levels in smokers that did not reach statistical significance. The limited number of available studies, together with their methodological and clinical heterogeneity, may have contributed to the lack of statistical significance, and thus, additional well-designed studies are needed to clarify the potential association between smoking status and circulating sPD-L1 levels.

Regarding histology, several cohorts showed a tendency toward higher sPD-L1 levels in adenocarcinoma (24, 26), while only one study by Castello et al. reported a statistically significant difference between squamous cell carcinoma and adenocarcinoma (45.28 pg/mL vs. 25.68 pg/mL, p = 0.048; **Table 2**) (22). Even if it was not determined in every study, mutational status plays an important role in treatment choice and response to immunotherapy. It is well known that a patient with an EGFR mutation or an ALK fusion will have a poor response to immunotherapy (45). Multiple original studies included patients with confirmed driver mutations who were treated with immune checkpoint inhibitors (ICIs), with EGFR mutations being the most common, ranging from 2% (29) to 16% of the study population (2). Therefore, a patient with this profile may have a poorer prognosis, with shorter progression-free survival PFS and OS (45). Despite several studies showing no association between sPD-L1 and tissue PD-L1, Murakami et al. reported higher sPD-L1 levels in patients with PD-L1 TPS ≥ 50% (77.7 ± 28.9 pg/mL, p = 0.0101) (2). Moreover, although sPD-L1 is considered a soluble form derived from tumor cell PD-L1 through various mechanisms, Teramoto et al. and Mildner et al. observed an increase in circulating sPD-L1 levels after tumor resection, suggesting that additional factors may contribute to sPD-L1 production in peripheral blood (9, 21).

### 4.3 Baseline versus on-treatment sPD-L1

The timing of sPD-L1 assessment is another important aspect to consider when interpreting its prognostic value. In our meta-analysis, the proposed cut-off was derived exclusively from baseline, pre-treatment samples and should therefore be interpreted within this context. However, studies evaluating serial sPD-L1 measurements during immunotherapy have yielded different prognostic associations. Oh et al. found that patients with a >100% increase in sPD-L1 after initiation of immune checkpoint inhibitor therapy had significantly longer PFS and OS (Oh et al., 2021). Similarly, Yi et al. observed an early increase in sPD-L1 following anti-PD-1 therapy, which was followed by a decline in responders, whereas persistently elevated levels were observed in non-responders (28). Comparable findings have also been reported in melanoma, where longitudinal changes in circulating soluble immune checkpoint molecules were associated with treatment outcomes during anti-PD-1 therapy (46). These observations suggest that the prognostic significance of sPD-L1 depends not only on its circulating level but also on the timing of sample collection. While baseline measurements provide prognostic information before treatment initiation, serial measurements may offer additional insight into treatment-related changes. Whether different cut-off values are needed for baseline and on-treatment sPD-L1 measurements remains to be determined.

### 4.4 Confounding factors influencing survival outcomes in NSCLC patients

Several confounding factors, including gender, smoking status, and tumor burden, can influence survival outcomes in NSCLC patients. Among those, gender has emerged as a significant confounder in survival analyses of non-small cell lung cancer (NSCLC), affecting both progression-free survival (PFS) and overall survival (OS). Multiple studies have shown that female patients often experience better survival outcomes than males, independent of treatment modality, disease stage, or histologic subtype (47–49). For example, men with NSCLC had significantly lower survival (HR 1.493, 95% CI 1.238–1.800, p < 0.001) even after adjustment for relevant clinical variables, and this remained significant in the matched cohort (HR 1.339, 95% CI: 1.075–1.667, p = 0.009). Similarly, within the same cohort, men with stage IV adenocarcinoma demonstrated significantly worse survival after adjusting for other clinical factors (HR 1.493, 95% CI: 1.238–1.800, p < 0.001) and in the matched cohort (HR 1.339, 95% CI: 1.075–1.667, p = 0.009) (48).

Smoking status is an important confounder in the assessment of PFS and OS in NSCLC patients, as it influences both tumor biology and treatment response. Immunotherapy has been shown to significantly improve OS and PFS compared with chemotherapy or placebo in smokers (OS: HR 0.76 [0.69–0.83], p < 0.00001; PFS: HR 0.65 [0.56–0.75], p < 0.0001), whereas benefits were smaller or not statistically significant in non-smokers (OS: HR 0.91 [0.78–1.06], p = 0.25; PFS: HR 0.68 [0.45–1.03], p = 0.07). In the same cohort, these trends were consistent across first-line and later-line treatments, as well as in patients with non-squamous NSCLC and smokers receiving immunotherapy experienced longer OS (HR 0.86 [0.75–0.99], p = 0.04) and PFS (HR 0.69 [0.60–0.81], p < 0.0001), along with a higher objective response rate (ORR: 1.20 [0.94–1.53], p = 0.15), compared with non-smokers. (50). A separate study found that former smokers had the longest median PFS (17.0 months, p < 0.001), with heavy smokers also showing favorable outcomes (PFS 15.9 months, p = 0.001). Multivariate analysis further indicated that former heavy smokers receiving immunotherapy were independent predictors of improved PFS (HR 0.75, 95% CI: 0.57–0.99, p = 0.04), emphasizing the need to account for smoking history in survival assessments. (51). Baseline tumor volume is a strong predictor of outcomes in advanced NSCLC. In the immunotherapy era, Uehara et al. reported that patients with high baseline tumor burden had significantly worse outcomes under PD-1/PD-L1 blockade, with median PFS of 11.8 vs. 2.4 months in the small versus large tumor groups (HR 0.38; 95% CI: 0.22–0.65; p < 0.001). Baseline tumor burden also remained an independent predictor of both PFS and OS on multivariable analysis (52). Beyond anatomical size, volumetric and metabolic parameters provide additional prognostic value. FDG-PET/CT studies have shown that high pre-treatment metabolic tumor volume and total lesion glycolysis (TLG) are strongly linked to poor survival. In a multicenter analysis of stage I–IV NSCLC, univariate analysis revealed a significant association between overall survival (OS) and clinical stage, with HR of 1.04 for stage II (p = 0.946), 3.60 for stage III (p = 0.001), and 4.00 for stage IV (p = 0.013) compared with stage I (53). More recent PET-based studies indicate that metabolic tumor volume provides additional prognostic information beyond TNM staging and RECIST measurements, particularly in cohorts consisting exclusively of metastatic patients (54).

These findings highlight the importance of considering confounding factors such as gender and smoking history when assessing progression-free and overall survival in NSCLC patients receiving immunotherapy. This consideration is particularly critical in studies involving soluble biomarkers, as their levels and prognostic value may be modulated by these patient-specific characteristics, potentially influencing the interpretation of treatment outcomes.

## 5 Conclusions

In conclusion, this meta-analysis suggests that sPD-L1 may serve as both a prognostic and predictive biomarker for survival in lung cancer patients undergoing various treatments, including immunotherapy, chemotherapy, radiotherapy, or surgery (**Figure 7**). Specifically, elevated plasma or serum sPD-L1 levels were significantly associated with poorer PFS and OS. Further studies are needed to establish optimal cut-off values and validate its utility across different treatment modalities. Moreover, incorporating confounding factors such as gender, smoking status, and tumor burden alongside soluble immune checkpoint markers (e.g., sPD-L1, sPD-1) into predictive models may improve prognostic accuracy.

## Data Availability

The original contributions presented in the study are included in the article/**Supplementary Material**. Further inquiries can be directed to the corresponding author.
