# Supplementary material for: Soluble immune checkpoints in lung cancer: linking prognostic signatures to immunotherapy response – a meta-analysis
Source: Front Immunol. 2026 Jul 29;17:1896427. doi: 10.3389/fimmu.2026.1896427 (PMC13462062; doi:10.3389/fimmu.2026.1896427)
Supplement: Supplementary file 1 [file Table1.docx]

**Supplementary Table 1.** Measurement assays used to analyse circulating levels of soluble immune checkpoint molecules.

| **Study** | **Measurement Assay** |
| --- | --- |
|  |  |
| **sPD-L1** | |
| Zhao et al., 2017 | ELISA (PDCD1LG1; USCNLife Science, Wuhan, China) |
| Okuma et al., 2017 | ELISA (PDCD1LG1; Cloud-Clone Corp, Houston, TX, USA) |
| Okuma et al., 2018 | ELISA (PDCD1LG1; Cloud-Clone Corp, Houston, TX, USA) |
| Costantini et al., 2018 | ELISA (#ab214565 Human PD-L1 [28-8]; Abcam, Cambridge, UK) |
| Castello et al., 2020 | ELISA (PD-L1/CD274 Quantikine ELISA; R&D Systems, Inc, Minneapolis, MN, USA) |
| He et al., 2020 | ELISA (Suzhou Bright Scistar Biotechnology Company Ltd., Suzhou, China) |
| Murakami et al., 2020 | ELISA (#DB7H10; R&D Systems, Inc, Minneapolis, MN, USA) |
| Mazzaschi et al., 2020 | ELISA (#DB7H10; R&D Systems, Inc, Minneapolis, MN, USA) |
| Tiako Meyo et al., 2020 | ELISA (#SEA788Hu; Cloud-Clone Corp, Houston, TX, USA) |
| Oh et al., 2021 | ELISA (#BMS2212; Invitrogen, Vienna, Austria) |
| Himuro et al., 2023 | ELISA (PD-L1/B7-H1 Quantikine ELISA Kit; R&D Systems, Minneapolis, MN, USA) |
| Chmielewska et al., 2023 | ELISA (#BMS2327; ThermoFisher, Waltham, USA) |
| Hayashi et al., 2024 | HISCL system |
| F.O.Milder et al., 2024 | ELISA (#SEA788Hu; Cloud-Clone Corp, Houston, TX, USA) |
| Han et al., 2024 | ELISA (#XG-K3016; Bright Scistar Anitbody Biotech, Suzhou, China) |
| **sPD-L2** | |
| Constantini et al., 2018 | ELISA (#BMS2215; ThermoFisher Scientific, Waltham, USA) |
| Han et al., 2024 | ELISA (#XG-K3016; Bright Scistar Anitbody Biotech, Suzhou, China) |
| **sPD-1** | |
| Himuro et al., 2022 | ELISA (Human PD-1 ELISA Kit; RayBio, Norcross, GA) |
| Hayashi et al., 2024 | HISCL System |
| Tiako Meyo et al., 2020 | ELISA (#SEA751Hu; Cloud-Clone Corp, Houston, TX, USA) |
| **sCTLA-4** | |
| Hayashi et al., 2024 | HISCL System |
