# Supplementary material for: Soluble immune checkpoints in lung cancer: linking prognostic signatures to immunotherapy response – a meta-analysis
Source: Front Immunol. 2026 Jul 29;17:1896427. doi: 10.3389/fimmu.2026.1896427 (PMC13462062; doi:10.3389/fimmu.2026.1896427)
Supplement: Supplementary file 2 [file Table2.docx]

**Supplementary Table 2.** Main characteristics of the selected studies and patient information, including tumor stage, treatment, and potential confounders such as smoking history, ECOG performance status, mutational profile, and tumor histological subtypes.

| **Study** | **Region** | **Study design** | **Sample size** | **Stage** | **Treatment** | **Outcome** | **sPD-L1 Cut-Off** | **Tissue PD-L1** | **Endpoint** | **Median Follow-up (months)** | **ECOG** | **Smoking status** | **Histology** | **Driver mutations** | **Comments** |
| --- | --- | --- | --- | --- | --- | --- | --- | --- | --- | --- | --- | --- | --- | --- | --- |
| Zhao et al., 2017 | Japan | Prospective | 126 | IIIB | Radiotherapy | pre-treatment | 96.5 pg/mL | ND | OS | 25 | 0: n=50 (39.7%);  1: n=55 (43.6%);  2: n=21 (16.7%) | Current: n=69 (54.8%);  Former: n=53 (42.1%);  Nonsmoker: n=4 (3.1%) | ADC: n=57 (45.2%);  SCC: n=64(50.8%) | ND |  |
| Zhao et al., 2017 | Japan | Prospective | 86 | IIIB | Chemo-radiotherapy^a^ | pre-treatment | 89.6 pg/mL | ND | OS | 25 | 0: n=50 (39.7%);  1: n=55 (43.6%);  2: n=21 (16.7%) | Current: n=69 (54.8%);  Former: n=53 (42.1%);  Nonsmoker: n=4 (3.1%) | ADC: n=57 (45.2%);  SCC: n=64(50.8%) | ND |  |
| Okuma et al., 2017 | Japan | Prospective | 96 | IIIB-IV | Chemotherapy/ Best supportive care | pre-treatment | 7.32 ng/mL | ND | OS | 75 | 0–1: 54 (56.3%);  ≥2: 42 (43.7%) | No/light: n=36 (37.5%);  Previous/ heavy smoker: n=60 (62.5%) | NSCLC: n=81 (84.4%)  ADC: n=73(76.1%);  SCC: n=7(7.3%);  LC: n=1(1%)  SCLC: n=15(15.6%) | EGFR, ALK wild type: n=51 (69.9%);  EGFR: n=19 (26%); ALK: n=3 (4.1%) |  |
| Okuma et al., 2018 | Japan | Prospective | 39 | IV | Anti-PD-1^b^ | pre-treatment | 3.357 ng/mL | ND | PFS, OS | 14 | 0-1: n=15 (38.5%); ≥2: n=24 (61.5%) | No/light: n=11 (28.2%);  Heavy: n=28 (71.8%) | NSCLC: n=2 (5.1%) ADC: n=28 (71.8%) SCL: n=7 (17.9%)  LC: n=2 (5.1%) | EGFR and ALK wild type: n=26 (81.3%);  EGFR: n=5 (15.6%);  ROS1: n=1 (3.1%) |  |
| Costantini et al., 2018 | France | Prospective | 43 | I-IV | Anti-PD-1^b^ | pre- and post-treatment | 33.97 pg/mL | Available in 34 of 43 patients (79%), with 24 (71%) positive | PFS, OS | 16.3 | 0-1: n=25 (58%); 2: n=17 (40%); 3: n=1 (2%) | Current: n=14 (33%);  Former: n=24 (55%); Nonsmoker: n=5 (12%) | ADC: n=28 (65%); SCC: n=9 (21%)  LC: n=4 (9%);  Sarcomatoid: n=1 (2%)  Clear cell: n=1 (2%) | EGFR, ALK wild type: n=23 (53%);  EGFR:n=1 (2%);  KRAS: n=15 (35%);  HER 2 n=1 (2%), BRAF n=3 (7%). |  |
| Ando et al., 2019 | Japan | Retrospective | 12 | IIIA-IVB | Anti-PD-1 | pre- and post-treatment | 347.37 pg/mL | >50% (n=9); 10–20% (n=1); 20–30% (n=1) | OS | N/A | ND | ND | ND | ND | A cohort comprising various malignancies^c^ |
| He et al., 2020 | China | Retrospective | 88 | IA-IIIB | Surgical intervention | pre-treatment | 3.4 ng/mL | ND | OS | 67 | ND | Yes: n=55 (62.5%); No: n=33 (37.5%) | ADC: n=38 (43.2%); SCC: n=50 (56.8%) | ND |  |
| Murakami et al., 2020 | Japan | Retrospective | 233 | III-IV | Anti-PD-1 | pre-treatment | 90 pg/mL | >50% (n=79;34%);  1-49% (n=44; 19%);  <1% (n=33; 14%) | PFS, OS | 36 | 0-1: n=211: (91%)  2: n=22 (9%) | Current/former smoker: n=179 (77%); Never: n=54 (23%) | SCC: n=52 (22%); NonSCC: n=181 (78%) | EGFR wild-type: n=135 (58%); EGFR: n=37 (16%) |  |
| Mazzaschi et al., 2020 | Italy | Prospective | 109 | IIIB-IB | Anti-PD-1, Anti-PD-L1^d^ | pre-treatment | 113 pg/mL | <1% (negative); 1–49% (intermediate); 50–100% (high), counts not reported | PFS, OS | 17.3 | 0-1: n=95 (87%)  2: n=14 (13%) | Smokers: n=36 (33%); Ex-Smokers: n=48 (44%); Non-Smokers: n=25 (23%) | SCC: n=32 (30%); ADC: n=70 (64%); NSCLC NOS: n=7 (6%) | KRAS: n=19 (17%); EGFR: n=6 (5%); ALK: n=1 (1%); Other: n=5 (4%) |  |
| Tiako Meyo et al., 2020 | France | Prospective | 51 | IV | Anti-PD-1^b^ | pre- and post-treatment | 57 pg/mL. | 0% n=14 (27.5%); 1–4% n=3 (5.9%); 5–9% n=6 (11.7%); 10–24% n=5 (9.8%); 25–49% n=3 (5.9%); ≥50% n=9 (17.6%); Unknown n=11 (21.6%) | PFS, OS | 26 | 0: n=1 (2%)  1: n=29 (56.8%)  2: n=19 (37.2%)  3: n=2 (4%) | Active: n=12 (23.5%);  Former: n=37 (72.5%); Never: n=2 (4%) | ADC: n=40 (78.4%) SCC: n=11 (21.6%) LC: n=0 (0%) | EGFR: n=2 (4%); ALK: n=2 (4%); KRAS: n=12 (23.5%); Other:n=4 (7.8%) |  |
| Castello et al., 2020 | Italy | Retropective | 20 | III-IV | Anti-PD-1, Anti-PD-1 and Anti-CTLA-4 | pre- and post-treatment | 27.22 pg/mL | ND | PFS, OS | 10.3 | 0: 60% | Smokers: 80% | ADC: 70% | ND | Embedded in the prospective study NCT03563482 |
| Oh et al., 2021 | Korea | Prospective | 50 | IV | Anti-PD1,  Anti-PD-L1,  Anti-CTLA-4 | pre-treatment | 11.0 pg/mL | Negative/weak: 43; Moderate/strong: 25; Unknown: 60 (tumor type not specifiend | PFS, OS | 48 | 0: n=27 (21.1%); 1: n=98 (76.6%); 2: n=1 (0.8%) | ND | NSCLC: n=50 (39.1%); SCLC: n=14 (10.9%) | ND | A cohort comprising various malignancies ^e^ |
| Himuro et al., 2023 | Japan | Prospective | 189 | III-IV | Anti-PD-1, Anti-PD-L1^d^ | pre-and post-treatment | 55.3 pg/mL, 63.7 pg/mL^f^ | 0-49% n=54 (44.3%);  50-100% n=55 (45.1%)  Unknown n=13 (10.7%) | PFS | N/A | 0-1: n=58 (48.3%)  2-3: n=62 (51.7%) | Former: n=94 (78.3%); Never: n=26 (21.7%) | SCC: n=32 (26.7%);  NonSCC: n=88 (73.3%) | Wild type: n=104 (86.7%); EGFR or ALK: n=16 (13.3%) |  |
| Himuro et al., 2023 | Japan | Prospective | 189 | III-IV | Anti-PD1,  Anti-PD-L1^d^ | pre-and post-treatment | 92.9 pg/mL, 99.8 pg/mL^f^ | 0-49% n=54 (44.3%);  50-100% n=55 (45.1%)  Unknown n=13 (10.7%) | OS | N/A | 0–1: n=59 (48.4%); 2–3: n=63 (51.6%) | Former: n=95 (77.9%); Never: n=27 (22.1%) | SCC n=32 (26.2%); NonSCC-: n=90 (73.8%); | Wild type: n=106 (86.9%); EGFR or ALK: n=16 (13.1%) |  |
| Botticelli et al., 2022 | Italy | Prospective | 36 | IV | Anti-PD-1 | pre-treatment | 38.4 pg/mL | ND | PFS, OS | 40 | ND | ND | ND | ND | A cohort comprising various malignancies ^g^ |
| Chmielewska et al., 2023 | Poland | Retrospective | 120 | IIIB-IV | Anti-PD-1, Anti-PD-L1^d^ | pre-treatment | 20 pg/mL | ≥ 1% n=95 (81.2%);  <1% n=22 (18.8%) | PFS, OS | 42.5 | ND | ND | SCC: n=50 (41.7%); NonSCC: n=70 (58.3%) | Wild type: n=120 (100%) |  |
| Teramoto et al., 2023 | Japan | Prospective | 69 ^h^ | IA-IV | Surgical intervention | pre- and post-treatment | 80 pg/mL | Determined, values not reported | RFS | 61 | ND | Determined, values not reported | Determined, values not reported | ND | RFS=Surrogate marker for PFS |
| Yi et al., 2023 | China | Prospective | 39 | IIB-IIIC, IV | Anti-PD-1 | pre- and post-treatment | 280.64 pg/mL | ≤0.5: n=15; >0.5: n=19 | PFS | N/A | ND | Never: n=10 Smoker: n=29 | ADC: n=20 SCC: n=19 | ND |  |
| Hayashi et al., 2024 | Japan | Prospective and Retrospective | 50+262 ^i^ | Advanced | anti-PD-1^b^ | pre-treatment | 205 pg/mL | 0% n=16;  1–49% n=17;  >50% n=17;  Unknown: n=0 | PFS | 13.2 | 0: n=18 (36%)  1: n=32 (64%)  2: n=0 | Current/former: n=27 (54.0%); Never: n=23 (46.0%) | ADC: n=36 (72.0%) SCC: n=12 (24.0%) ADC-SCC: n=1 LC: n=1 | EGFR: n=12 (24.0%);  ALK: n=2 (4.0%); ROS1: n=0 (0%) |  |
| Han et al., 2024 | China | Prospective | 95 | I-IV | Diverse therapeutic approaches ^j^ | pre- and post-treatment | 713.75 pg/mL | ND | OS | 24 | ND | Determined, values not reported | Determined, values not reporter | Determined, values not reported |  |
| F.O. Mildner et al., 2024 | Austria | Retropective | 74 | IA-IIIB | Surgical intervention | pre- and post-treatment | 1000 pg/mL | Determined, values not reported | RFS, OS | 45 | ND | Current/former smoker: n=59;  Never: n=5;  Unknown: n=10 | ADC: n=58;  SCC: n=11;  Unknown: n=10 | ND | RFS=Surrogate marker for PFS |
| Brun et al., 2024 | Denmark | Retropective | 80 | II-IV | Anti-PD-1, Anti-PD-L1^d^ | pre- and post-treatment | 52 pg/mL | <50% n=17 (21%)  ≥50% n=63 (79%) | PFS, OS | 24 | 0: n=44 (55%)  1-2: n=36 (45%) | Active: n=20 (25%);  Previous: n=55 (69%);  Never: n=2 (3%) | ADC: n=56 (70%) SCC: n=17 (21%) Other: n=7 (9%) | ND |  |

**Abbreviations**: ND: not determined, N/A:not announced, NSCLC: non-small cell lung cancer, SCLC: small cell lung cancer, ECOG: Eastern Cooperative Oncology Group, ADC: adenocarcinoma, SCC: squamous cell carcinoma, LC: large cell carcinoma, EGFR mutation: epidermal growth factor receptor mutation, ALK: anaplastic lymphoma kinase rearrangements:, ROS1 = ROS proto-oncogene 1 rearrangements:, KRAS = Kirsten rat sarcoma viral oncogene homolog mutation**,** BRAF = B-Raf proto-oncogene serine/threonine kinase mutation, HER = human epidermal growth factor receptor mutation, NSCLC NOS = non-small cell lung cancer, not otherwise specified, ADC-SCC: adenosquamous

NSCLC NOS = non-small cell lung cancer, not otherwise specified

Anti-CTLA-4 therapy: monoclonal antibodies targeting the CTLA-4 receptor, such as Ipilimumab.

Anti-PD-1 therapy: monoclonal antibodies targeting the PD-1 receptor, such as Nivolumab, Pembrolizumab.

Anti-PD-L1 therapy: monoclonal antibodies targeting the PD-L1 receptor, such as Atezolizumab, Durvalumab.

**^a^** All patients received daily radiotherapy (2.0–3.8 Gy/fraction, 6–25 MV) with concurrent weekly carboplatin (AUC 2) and paclitaxel (45 mg/m²) for 6 weeks;

**^b^** Anti-PD-1 refers exclusively to Nivolumab in this study;

**^c^** NSCLC, Gastric cancer, Bladder cancer;

^d^ Anti-PD-L1 refers exclusively to Atezolizumab in this study;

^e^ NSCLC, SCLC, RCC, HNSCC, Urothelial carcinoma (UCC), Salivary gland cancer and others;

^f^ First cut-off is for pre-treatment evaluation; Second cut-off is for post-treatment evaluation;

^g^ NSCLC; Uveal Melanoma; Renal Cell Carcinoma (RCC); Head and Neck Squamous Cell Carcinoma (HNSCC);

^h^ Out of 69 patients, 63 had NSCLC; plasma sPD-L1 was assessed preoperatively, with 55 radical surgery cases included in the final analysis;

^i^ Cohort B (n=149), Cohort C (n=28), Cohort D (n=42), Cohort E (n=43);

^j^ Among 55 lung cancer patients: 35 received monotherapy (chemotherapy = 14, radiotherapy = 9, targeted therapies= 18, anti-PD-1= 5, palliative care = 9), 20 combination therapy, and 20 periodic reviews.
